# Pd-Catalyzed Cross-Couplings: On the Importance of the Catalyst Quantity Descriptors, mol % and ppm

**DOI:** 10.1021/acs.oprd.2c00051

**Published:** 2022-07-11

**Authors:** Christopher S. Horbaczewskyj, Ian J. S. Fairlamb

**Affiliations:** University of York, Heslington, York, North Yorkshire, YO10 5DD, United Kingdom

**Keywords:** palladium, ppm palladium, cross-coupling, Suzuki−Miyaura, Kumada, Negishi, Stille, Heck, Sonogashira, cyanation, direct arylation, Buchwald−Hartwig amination cross-coupling, quantification, synthesis, catalysis

## Abstract

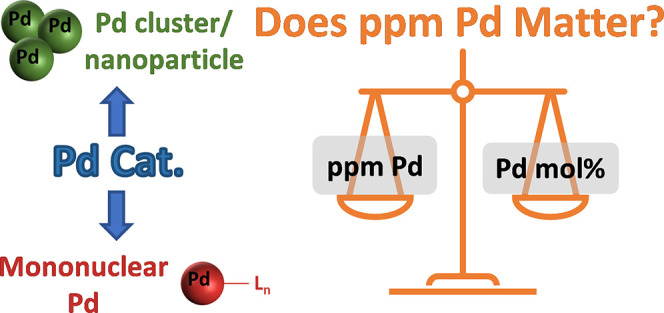

This Review examines parts per million (ppm) palladium concentrations in catalytic cross-coupling reactions and their relationship with mole percentage (mol %). Most studies in catalytic cross-coupling chemistry have historically focused on the concentration ratio between (pre)catalyst and the limiting reagent (substrate), expressed as mol %. Several recent papers have outlined the use of “ppm level” palladium as an alternative means of describing catalytic cross-coupling reaction systems. This led us to delve deeper into the literature to assess whether “ppm level” palladium is a practically useful descriptor of catalyst quantities in palladium-catalyzed cross-coupling reactions. Indeed, we conjectured that many reactions could, unknowingly, have employed low “ppm levels” of palladium (pre)catalyst, and generally, what would the spread of ppm palladium look like across a selection of studies reported across the vast array of the cross-coupling chemistry literature. In a few selected examples, we have examined other metal catalyst systems for comparison with palladium.

## Introduction

Cross-coupling reactions are a central cog in the chemical machinery needed to access complex targets such as natural products,^1−3^ pharmaceuticals,^4−6^ or agrochemical chemical^7−9^ structures, found in both academic and industrial settings.^10^ Significant research has been aimed at making each (cross-coupling) reaction more efficient and environmentally friendly. This can be accomplished using less hazardous solvents, reducing the total reaction volume, considering the total energy demand and carbon sustainability, changing the (pre)catalyst (including catalytic design and engineering),^11,12^ or reducing the amount of (pre)catalyst used.^11,13^ We use the term (pre)catalyst to indicate that a chemical change needs to occur in the initial stages of the reaction, whether it be an intentional change or not, for the delivery of the active catalyst species.

The most common (pre)catalysts applied to these reactions are oftentimes based on precious metals with Pd being the metal catalyst of choice in many applied processes. Pd catalysts typically outperform more abundant earth metals such as Ni, Fe, Mn, Cu, or Co although the gap is narrowing.^14−16^ What is clear is that Pd cost is emerging as a significant barrier, particularly for the agrochemical sector. While great progress with earth-abundant metal catalysts has been made, the current go-to catalytic processes often still involve Pd. One of the issues is that Pd is embedded in patents or current processes, as a keystone in a particular route that cannot be (easily) changed, although adaptions might be possible. On the other hand, the wider field working on earth abundant catalysis^17−19^ arguably needs to grapple with high metal catalyst loadings that are typically needed for applied transformations (that is accessing more complex synthetic targets). These high loadings could prove problematic at later process stages during separation and increase the risk of downstream contamination. Associated are the challenges in improving catalytic performance, particularly from an industrial practical perspective, which our group has experienced working in the field of manganese(I) C–H bond functionalization catalysis.^20−23^

For the field of Pd cross-coupling chemistry, understanding and harnessing catalyst speciation is important. As an example, by varying the amount of PPh_3_ ligand with Pd(OAc)_2_, a ubiquitous (pre)catalyst, one can alter the reaction outcome of a Suzuki–Miyaura cross-coupling (SMCC) involving a dihalogenated pyridine, switching arylation site-selectivity from C2 to C4.^24^ The addition of two equivalents of PPh_3_ with Pd(OAc)_2_ forms a Pd^I^ species, which then forms a highly active competent cross-coupling Pd_3_-cluster catalyst upon addition of R-X.^24−26^ Such changes in Pd catalyst speciation, while complicated and oftentimes confounding, offer new opportunities in catalyst design, potentially allowing low catalyst loadings (and low ppm Pd) to be accessed more readily. The exploitation of Pd catalyst speciation in altering established reaction outcomes represents a fascinating opportunity.

**Scheme 1:**
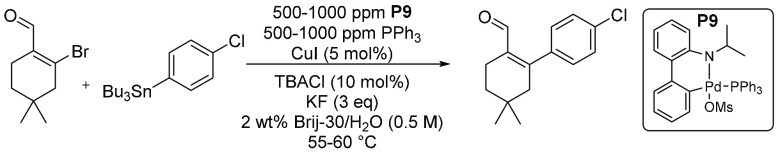
Stille Reaction in Water Using a PPh_3_-Based Palladacycle (4.2 ppm)^27^

Recent work, described in several insightful papers,^27−45^ outlines the use of “ppm level” palladium (“sustainable levels”) as one of several propositions for the improvement of various catalytic reactions. One study showed that a Stille reaction employing micelles^27^ operated at low Pd (pre)catalyst loadings at the sub-ppm level (Scheme 1). Each stock solution (500 or 1000 ppm), for both catalyst and ligand, was prepared before an aliquot (25 μL) of each was added to a vial containing each reaction component, giving a mean Pd concentration of 4.2 ppm in the final mixture.

**Scheme 2:**
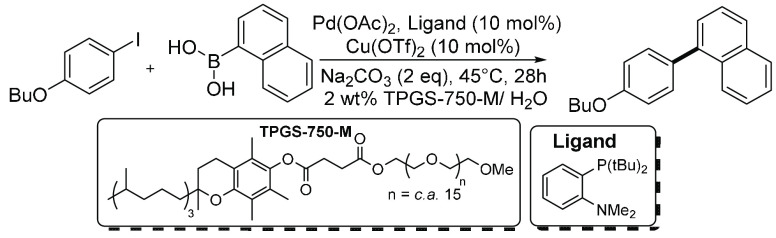
SMCC Reaction Using 200 ppm Pd and Cu(OAc)_2_

Furthermore, a dual Cu and Pd catalyst system was used for a SMCC reaction (Scheme 2).^34^ For optimization, the following conditions were employed: water, mild conditions, catalyst recycling, and the use of “ppm levels” of Pd. Pd(OAc)_2_ (200 ppm) was added to Cu(OTf)_2_ and ligand (0.5 mmol each). This equates to ∼23 ppm, taking into consideration all other reagents and solvent (1.1 mL, 0.145 mol) added, which is an important detail. While this reaction Pd catalyst ppm value is low, several of our subsequent analyses (i.e., historical studies, vide infra), around 20% of the papers examined, employed a similar concentration of Pd (pre)catalyst or lower; i.e., the amount of reaction moles is not meaningful outside the context of the scale of a specific reaction.

**Scheme 3:**
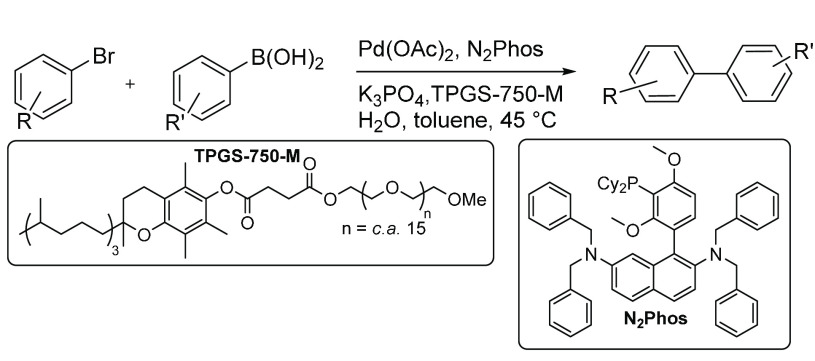
SMCC Reaction of Aryl Bromide and Aryl Boronic Acid Using Pd(OAc)_2_ and N_2_Phos in Water and Toluene^39^

Another SMCC reaction^39^ exploited a newly designed ligand (N_2_Phos) and “ppm level” Pd to improve the reaction conditions (Scheme 3). A stock Pd catalyst solution of ∼2500 ppm (equivalent to ∼1000 ppm) enabled the use of 0.1 mol % Pd catalyst in SMCC reactions (1 ppm).

In a further SMCC reaction, improvements were made using a palladacycle (pre)catalyst.^44^ The reaction used a stock catalyst/ligand solution made up to 10 000 ppm Pd before being added to the reaction, giving an overall Pd concentration of 300 ppm. Neither the molar ratio nor the mass used to make up the stock solution were stated. We were able to examine the experimental details within the supporting information and determine that the reaction was performed using 232 ppm, which is similar from the 300 ppm reported (based on mass).

Generally, the sustainability of a process will largely depend on the scale of operation. Use of “ppm level” palladium (in isolation) does not necessarily describe a sustainable process. For that, additional context is required. For research groups, small scale operations will use comparatively low levels of palladium (regardless of using “ppm level” palladium terminology) and will continue to be sustainable, particularly if waste palladium is collected and regenerated, while large scale Pd catalysis operations may not be sustainable unless the utilization of Pd aligns with production supply and demand.

Given that previous studies have outlined that Pd is used in ppm levels for efficient and selective catalysis, our interest in the wider field of Pd catalysis was stimulated. For this reason, it was decided that an expanded study into the levels of Pd used in cross-coupling reactions was significant to the broader field, as the use of ppm is not widely applied in this field (interestingly, ppm is more widely used in atmospheric chemistry to describe gaseous components).^46,47^ Thus, this Review aims to outline a new way of looking at catalytic reactions and detailing the concentration of Pd in the reaction system rather than focusing entirely on substrate to catalyst ratio (which is important in itself). The amount of catalyst used, in relation to the entire system, is key to understanding the reaction including all side reactions and side products, in addition to understanding the formation and evolution of Pd nanoparticles. Side products are rarely discussed, and many papers typically only mention the percentage yield of the major product. If side products are mentioned, then it is generally only the main side product, e.g., *E*/*Z* products during a Heck reaction. Side product distribution is complex in many catalytic reactions. The size,^48^ activity,^49^ and stability^50−52^ of nanoparticles is dependent on the concentration of metal added into a reaction. In addition, a change in the amount of (pre)catalyst will also change the concentration of active sites, which is important in the determination of the rate/TOF of fundamental kinetics, arguably making the concentration of catalyst a sensible metric, accompanying the ratio of (pre)catalyst to substrate (mol %). Palladium nanoparticle size formation can be varied by changing the initial concentration of palladium ions.^53^ Smaller nanoparticles are generally seen as more active as they have increased surface area and, depending on their shape, may have more active (defect) sites.^54^ For example, 1.8 nm palladium nanoparticles can efficiently catalyze a Suzuki–Miyaura cross-coupling reaction to gain almost 70% conversion.^55^

For this study, ppm was calculated as a molecular quantity; i.e., 1 ppm of catalytic Pd species is 1 molecule in every 1 × 10^6^ total molecules in the reaction. This metric was chosen because many of the papers examined failed to present the experimental data in a standardized and suitable format. Often, reaction “quantities” are provided as “eq” or only “mmol” with masses of reagents omitted either in part or completely. This made it difficult to calculate traditional ppm values, so molecular ppm was established as a fair comparison across all papers/studies.^56−64^ Using molecular quantities can enable easier visualization when considering catalytic mechanisms rather than a percentage of substrate moles. It may also be easier to relate to other molecular parameters, e.g., TOF, where the information is provided. Pd concentration can be shown in terms of molarity. However, this only relates palladium to the total solvent volume and does not account for any other species in the reaction mixture.

**Scheme 4:**
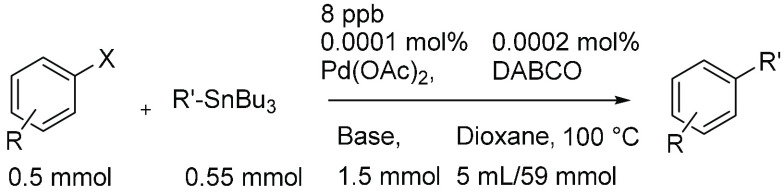
Stille Cross-Coupling Reaction of Aryl Halide and R-Tributyltin Using Pd(OAc)_2_ as Catalyst, DABCO as Ligand, Bu_4_NF as Activator, and Dioxane as Solvent For this reaction 0.0001 mol % Pd (pre)catalyst was used, correlating to 0.00816 ppm (8.16 ppb).

An example calculation for molecular ppm is given in Scheme 4.^65^

The total number of moles was calculated from each species including solvent.
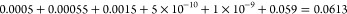


Then, the mole contribution of catalyst compared to the total number of moles was calculated:
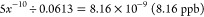


Multiplication by 1 × 10^6^ gives the molecular ppm of (pre)catalyst:
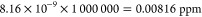


The data discussed in this Review is not an exhaustive picture of all cross-coupling reactions but draws on a selection (as representative examples) from each catalytic reaction across the literature. For each paper, the experimental procedure(s), tables/schematics, and associated supporting information (where available) were analyzed for the reaction details to calculate the total Pd catalyst ppm. Due to differences in data location and written form, accurate data extraction using scripted methods proved unfeasible. We further noted discrepancies between experimental procedures depending on journal (publisher) and/or author preference.

Experimental data was thus mined by manually searching, sorting, and assessing original published documentation, where the overall Pd (pre)catalyst ppm values were (re)calculated for a completed reaction system (including all reagents, substrates, catalyst, and solvents).

**Figure 1:**
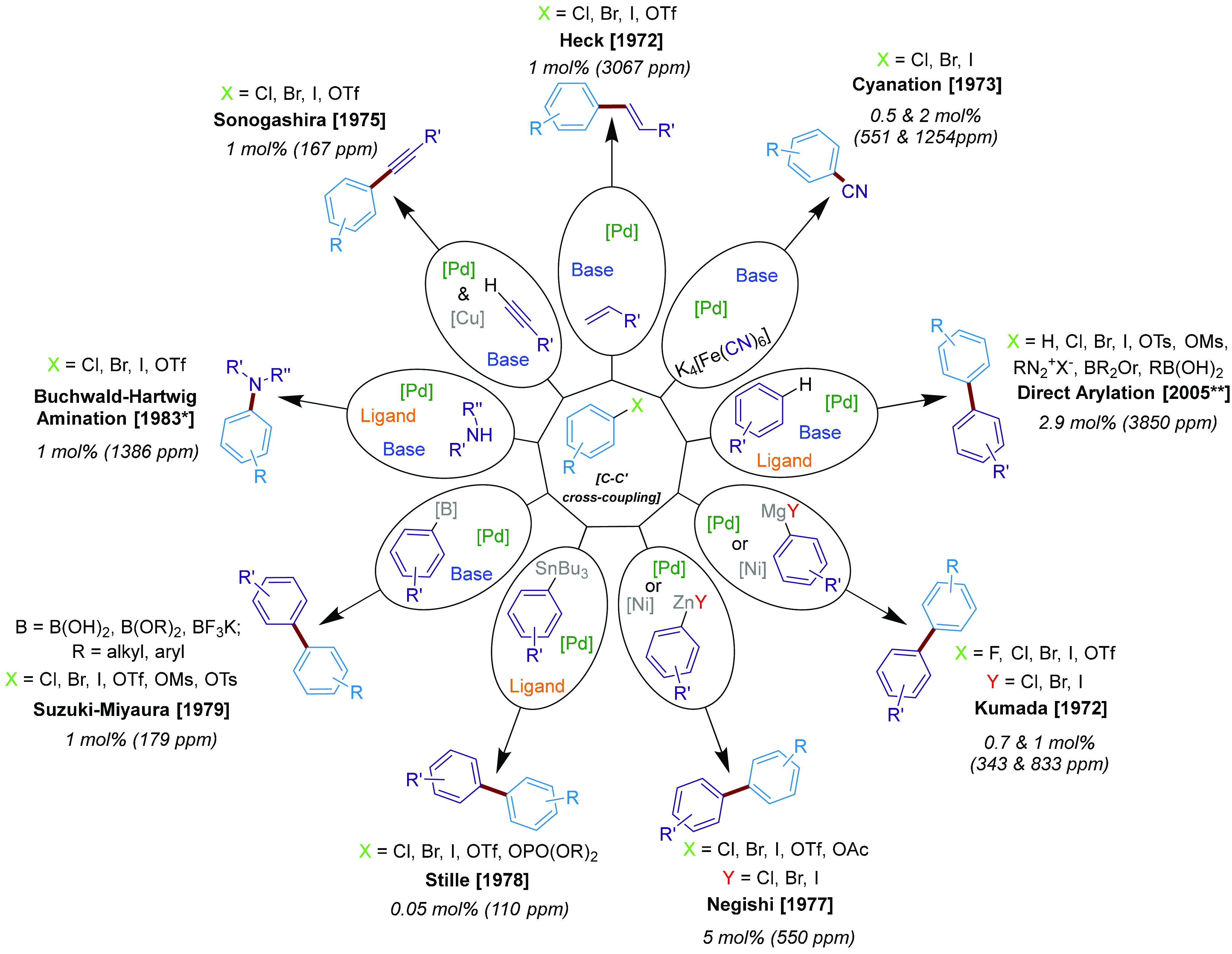
A summary diagram of all the Pd-catalyzed transformations (along with the year of first report, the mol %, and calculated molecular ppm) explored in this Review. *We recognize Migita and co-workers contributions to the discovery of the reaction of aminostannanes with organohalides.^64^ **The year of the first widely applied reactions.

Herein, we report an analysis of “ppm-Pd” literature for popular cross-couplings (Figure 1): Suzuki–Miyaura, Kumada–Corriu, Stille, Negishi, Sonogashira, Heck, cyanation, direct arylation, and Buchwald–Hartwig amination reactions. Calculations from a specific paper, for each reaction, relate Pd (pre)catalyst mol % to molecular ppm.

## Observations and Discussion

**Figure 2:**
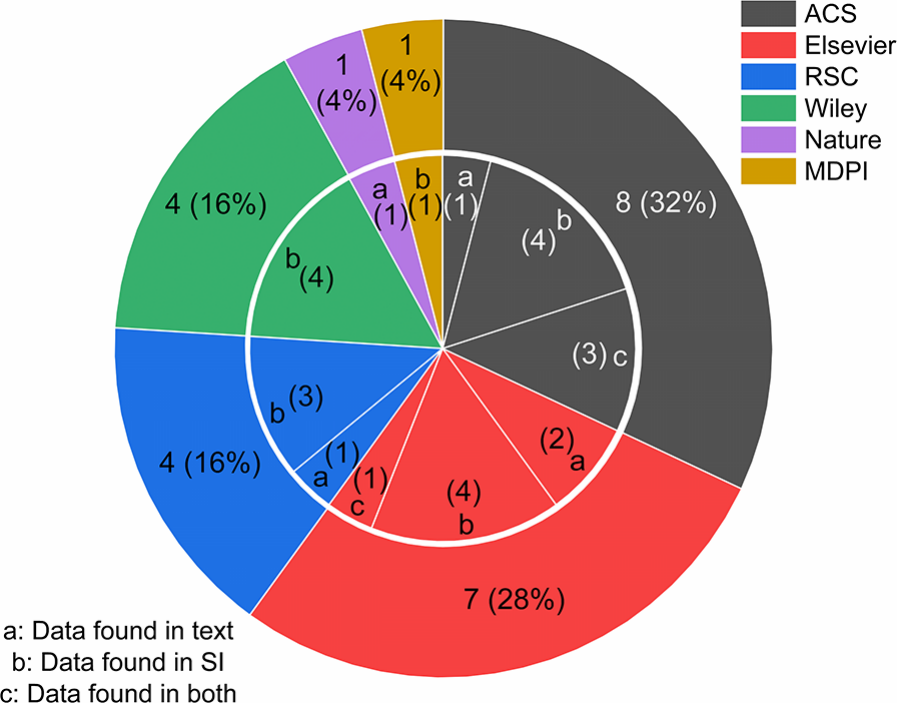
A pie chart outlining the total percentage of journals, from publishers of the papers used in this study. The inner chart shows the number of journals which fall into sources a, b, or c. Data in each paper could be found in either in the text (a) – including below figures/tables or in the papers’ experimental section (b), in associated supplementary information (c), or in both (a) and (b). For example: for the publisher ACS (gray section), ppm data came from 8 different journals (outer ring). Within those (inner ring) experimental data from one journal could be found in the paper’s text (a-1), four journals provided it in the associated supplementary information (b-4) and three journals with the information in the text and supplementary information (c-3).

There is a general expectation in modern chemistry journal articles to give the necessary experimental information in a presentable manner. Indeed, this is the case for many of the papers that we have analyzed. However, when undergoing a more detailed and thorough examination, discrepancies in reporting were noticeable. One of the main differences is the location of where the experimental data are reported: whether it is included in the main article text, e.g., in the main discussion text, a specific experimental section, a figure/table/scheme, or within an associated supporting information file (Figure 2). The journal often specifies where the data is placed and can be in a single place or multiple places. In addition to this, experimental data is reported using various unit formats, most often displayed in “mmol” but also reported using “eq” (equivalents) of each reagent with some authors reporting only in “g” (or “mL” for liquids). On rare occasions, some of these essential data were missing entirely (particularly in papers prior to the mid-2000s). From our perspective and following this study, we believe the following considerations could be made in reporting experimental data. If this process was standardized, then it could make future automated mining of data easier and more precise. We feel the subsequent points could be followed when writing experimental methods for journals.

- Include all data values: all reagent masses, equivalents, and molar quantities, catalyst/substrate ratio (mol %), volume (mL) if liquids present, ppm of catalyst in the global reaction accounting for all species, catalyst turnover numbers (TONs; defined as the number of moles of substrate converted to product relative to the number of moles of catalyst used), and catalyst turnover frequencies (TOFs; defined as the number of moles of substrate converted to product relative to the number of moles of catalyst used per unit time, in seconds).^66^
- While it is helpful to have the above details in the main text of a paper, e.g., below figures, tables, or schemes, or in the experimental section, this is not always necessary. An arguably easier searchable resource for these details is the associated paper supporting information or the appendix within an openly accessible PhD thesis.

An easier method to search for the necessary information is to compile a complete database of all reaction conditions and data values in a separate document, e.g., Excel or LIMS. This not only makes it easier to manually search but also ensures full accessibility for automated searches. A recent paper by Fitzner et al. highlighted how reaction databases can provide useful data about specific reactions, for example, in Buchwald–Hartwig cross-couplings. A total of 62 000 reactions in the patent literature (from CAS, Reaxys, and USPTO) were sorted, analyzed, and interpreted, showing skewed yield and an imbalance in reagent diversity. In addition, they found preferred solvents, bases, and ligands used for the Buchwald–Hartwig cross-coupling reaction.^67^

## Data Extraction and Organization

**Figure 3:**
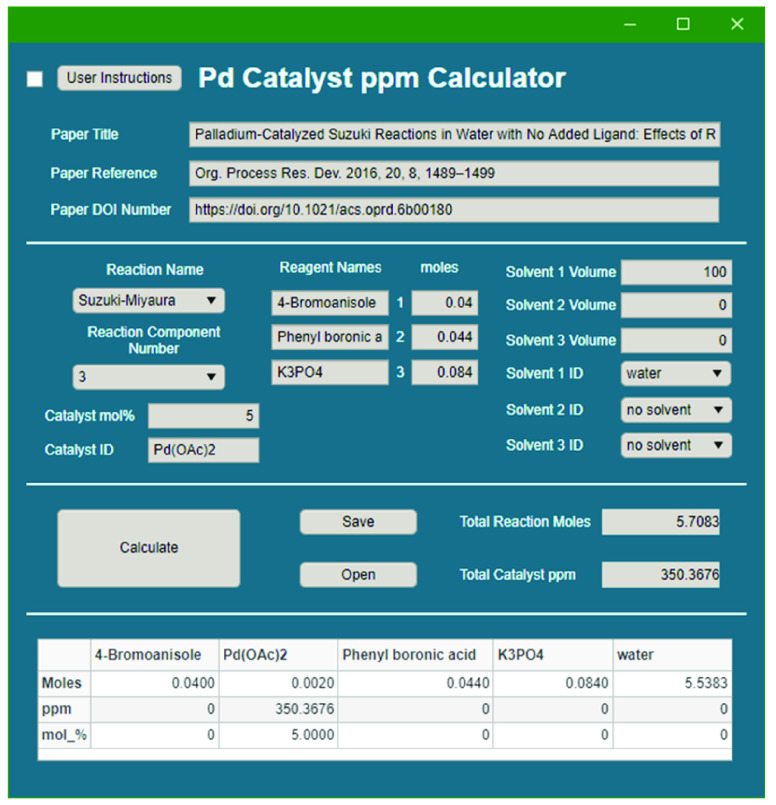
MATLAB app designed as part of this study to aid the user in sorting and calculating the Pd ppm levels in cross-couplings.

To efficiently aid data extraction, a program was written in MATLAB. The most time-consuming part of the data extraction process was finding solvent details to calculate the total moles of each reaction component. The process was simplified by creating a small database of solvent names, molecular weights, and densities that could be quickly and easily referenced to help calculate the final Pd ppm values. Initial coding made use of the MATLAB script functionality and only allowed the solvent reference and calculations to occur. This was subsequently upgraded to MATLAB app functionality (Figure 3) to sort the data into the required spreadsheets, calculate the required values, and allow greater flexibility of use (available as part of our Supporting Information).

The MATLAB app allows the user to select the reaction type, as a dropdown menu, before selecting the number of reagents (up to a maximum of five). Subsequently, the number of moles and reagent ID can then be entered. Alongside these, the catalyst ID, mol % value (in relation to the first reagent), and the solvent ID with its volume are chosen. Once these have been entered, the user may then calculate the in-reaction Pd ppm, which is displayed in the table alongside the total reaction moles. From here, the created table can be exported using the save functionality into a master Excel workbook where the data is stored, on the basis of the selected reaction name, and appended from the previous entry. The newly added data is also collated in the form of a list that autoupdates graphs in the Excel workbook. A DOI check for repeat papers also occurs to inform the user if the paper has been added previously. An open-source version of the app has been created using Python script (Supporting Information). This version includes slight improvements to the original MATLAB app (Supporting Information). An Excel file package for data storage is also part of the Supporting Information.

**Figure 4:**
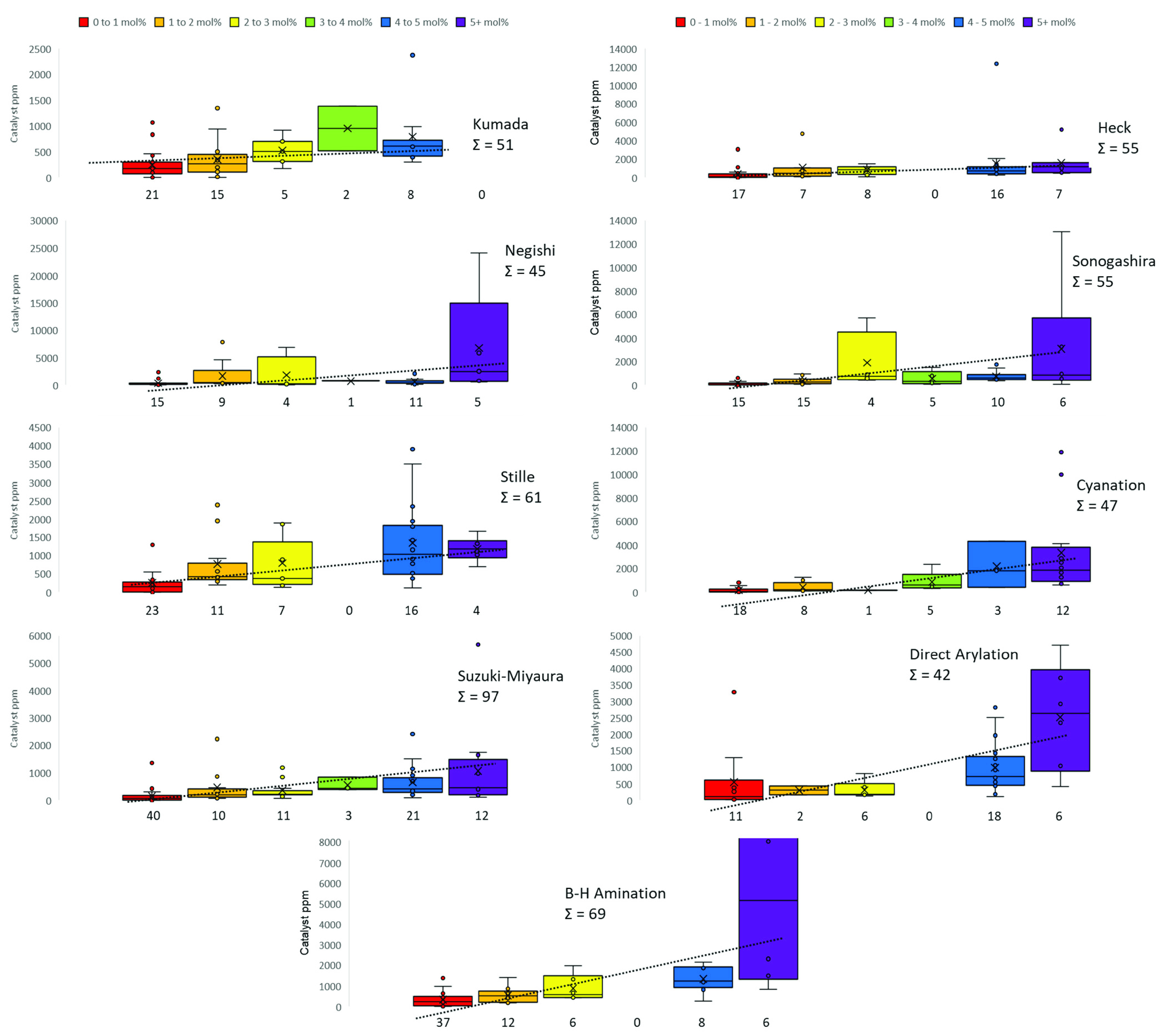
Stacked box and whisker plot outlining each named reaction during this study. Calculated catalyst ppm (molecular) is shown on each *y*-axis. Each box and whisker plot relates to a range of mol % values: red, 0–1; orange, >1–2; yellow, >2–3; green, >3–4; blue, >4–5; purple, 5+. The average ppm values in each category fit to the dotted line of best fit. Each box and whisker plot comprises the box, the interquartile range (middle 50% of data); central line, median value; whiskers, the total range of data; cross, mean value.

The data observations for each reaction can be made either individually or by comparison with each other. Note that most of the data presented here was gathered by searching the Web of Science for key terms related to each reaction (reaction name, palladium cross-coupling, palladium catalysis, palladium chemistry), being mindful of the author keywords and keywords plus sections. In addition, papers were chosen on the basis of the year published to try and formulate a range of dates from initial year of reaction publication (see Supporting Information). The Web site www.organic-chemistry.org also played a role in finding suitable, albeit recent, papers to be used in this study. The book *Applications of Transition Metal Catalysis in Drug Discovery and Development: An Industrial Perspective*(68) proved invaluable in identifying relevant named catalytic reactions from an industrial perspective, particularly those amenable to larger scale reactions. A minimum of 40 papers (for each reaction) was deemed to be representative due to time constraints (noting again that each paper was manually examined). This is not an exhaustive list, and this study has the potential to be ongoing. One difficulty we encountered was how to find papers that use a named catalytic reaction but do not mention so explicitly. We recognize that SciFinder, Reaxys, or similar structural databases might help in the future here. All data extracted from the papers are summarized in Figure 4, separated into each reaction, and represented as box and whisker plots for each mol % range with a line of best fit.

The numbers below each box indicate the total number of data points within each mol % range. For most reactions, there is a large skew in the data points toward lower mol % values, namely, 0–1 and 1–2 mol %. The 4–5 mol % category is common for Stille, Suzuki–Miyaura, and Heck reactions. The 5+ mol % category is used less often, which includes transition metals other than Pd, e.g., where Cu, Ni, and Fe are typically located (in this study). This can be ascribed (generally) to the lower catalytic activity of some of these systems, whereby the catalyst loading must be increased to see a similar performance level to that of Pd (note: again, we recognize there will be exceptions in the literature).

Concentration can have profound effects on reaction outcomes such as conversions and yields. Often, from the gathered data, solvent was the main constituent of each reaction, largely comprising 60–95% of the total molecular makeup for each reaction. This, as expected, had a large knock-on effect on catalyst ppm where a high catalyst mol % did not necessarily have large ppm values (i.e., the reaction was thus conducted using a low concentration of limiting reagent). There were a number of entries where solvent was not included as a reaction component and, as such, the total Pd molecular ppm is high. For example, 2433 ppm Pd was used in a solventless Negishi cross-coupling employing mechanochemically activated zinc.^69^

When it comes to determining the relative amounts of each component in a reaction, particularly in terms of catalysis, mole percent (mol %) or equivalents (eq or equiv) are most used where the catalyst is in a ratio with the main reaction substrate. This is useful for a few reasons:

(1) Provides the exact amount per mole of substrate
(2) Easy to relate to reaction relationships
(3) Easy for subsequent reaction calculations

However, other reaction components are less typically considered and may have effects on the overall reaction outcomes (e.g., product conversion, yield, selectivity). For higher catalyst loadings, we can anticipate that side reactions of the catalyst and/or ligands are more likely, i.e., leading to the generation of noticeable side products derived from the catalyst system. Reaction solvents typically make up the molecular bulk of the reactions. Indeed, how different solvents can change Pd catalyst speciation has been shown.^70,71^ Reagent solubility may play a role in why solvent is the molecular bulk of reactions or where reaction conditions are close to those described in the original manuscript procedure. Solvent selection is also a crucial factor to consider. In addition to Pd catalyst speciation changes, which may affect reaction pathways, different solvents can even increase or decrease reaction selectivity and/or reaction rate. Solvent molecules tend to be smaller (for the most part) than the reagent(s) or catalyst species, so the addition of 1 mL, for example, can add a significant number of extra molecules into the reaction medium, impacting the global reaction rates.

Experimental detail is most often recorded using mmol of each reagent along with the total volume of solvent (mL for small scale and L for large scale) added, which does not hint at the proportion of solvent in the system. Calculations using solvent density and molecular weight show that the largest proportion of molecules are derived from the solvent, depending on the solvent used. As an example, there is, on average, 108 times as many molecules of solvent as the limiting reagent (indicated by analysis of the Suzuki–Miyaura cross-coupling reaction data).

Regarding mol % usage, 5 mol % catalyst is often a popular choice. This is shown mainly in Suzuki–Miyaura (22%), Negishi (24%), Stille (26%), Heck (29%), and direct arylation (42%) reactions, where all have a high proportion of mol % values within the 4–5 mol % category (one example at 5 mol %). The remaining reactions: Sonogashira, Kumada, Buchwald–Hartwig amination, and cyanation (20% of papers examined) are within the 4–5 mol % range.

The employment of lower Pd catalyst loadings to these systems is potentially more susceptible to deactivation from uncontrollable contaminants in reagents/solvents/additives that are likely to interact with catalytic species. On the other hand, too much metal catalyst can make the removal from the final products more cumbersome. This is typically a problem on large scale where industry needs to develop new methods to reduce the level of Pd in active ingredients. It is necessary to remove Pd from final reaction mixtures or product compounds, where difficulties can occur depending on molecular structure and other parameters.^42,72,73^

There is a significant importance in the removal of Pd from reactions and during product purification. Effective Pd removal can be achieved using functionalized silica adsorbents or functionalized resins alongside more conventional workup/purification procedures. One such procedure by Magano et al. used SiliaMetS Thiol during the purification of a naphthalenopiperazine HCl salt. The crude product was contaminated with 1300–1600 ppm Pd. After treatment with SiliaMetS Thiol, the product was recovered in a 90% yield with only 2 ppm Pd. The reaction here used a Buchwald–Hartwig amination transformation with 2 mol % PdCl_2_(dtbpf) (7.6 ppm).^74^

Allmendinger and co-workers^75^ have shown how to prepare and apply different functionalized resins while scavenging heavy metals from reaction mixtures. The most effective consisted of a combination of silica resins and polyamines, mainly in apolar solvents. As scavenging brings an additional processing step, it must be compared to other alternatives to check viability. Reaction Pd can be removed by optimizing the reaction conditions, i.e., reduced Pd catalyst loading, or using other reaction processes such as product salt formation (if products are basic). It is worthwhile to examine multiple step reaction processes, as downstream transformations may assist in Pd removal.

Similarly, the use of polymer-supported ethylenediamine has been shown to be successful when removing residual Pd^0^ and Pd^II^ from a Suzuki–Miyaura cross-coupling reaction. In this work, the crude product contained 2000–3000 ppm palladium, which was initially reduced to 100–300 ppm using the supported scavenger and subsequently reduced again to <10 ppm via product salt formation.^76^

The effectiveness of metal scavengers has been shown to change under specific conditions. These relationships were determined using the design of the experiments (variables = temperature, scavenging time, amount of scavenger, and concentration of Pd in solution) with a Buchwald–Hartwig reaction. For example, at a higher temperature and longer scavenging time, the amount of scavenger needed to remove 90% of the reaction palladium increased to 1.2 mol equiv compared to 1 mol equiv when lower temperature and time were used. Pd scavenging from reactions is evidently a complex process where the initial amount of Pd used may play a role in the effectiveness of the scavengers.^77^

In general, reaction Pd ppm level does not appear to change with reaction scale and follows the same general trend as the rest of the data; higher mol % typically gives a higher molecular ppm. Often, equal reaction equivalents to those performed on small scale, which generally have low ppm levels, are used. From the larger scale reactions found, 75% were Suzuki–Miyaura with the final 25% consisting of Kumada (17%) and Stille (8%) reactions. The Kumada reactions made use of low catalyst loadings (0.1 and 1 mol %) while being performed on a 10 and 2 mol scale, respectively, where calculated ppm levels were 175 and 180. In contrast to this, a single large scale Stille reaction employed 5 mol % loading on a 1.8 mol scale, equating to a reaction ppm level of ca. 2345, which is significantly higher than the average ppm for all cross-coupling reactions (typically 815 ppm).

Extracted data from our study has shown that the most popular choices of (pre)catalyst are Pd(OAc)_2_, Pd_2_(dba)_3_, Pd(PPh_3_)_4_, and Pd(dppf)Cl_2_, which were used in ∼45% of all papers. Many entries in the <1 mol % category are often more specialized (pre)catalysts, e.g., [XantPhos Pd(allyl)]Cl or solid supported nanoparticles. Pd(OAc)_2_ was used in 83% of papers describing direct arylation.

The relation of reaction yield to molecular ppm is rather difficult to achieve from this data as reaction yield is affected by much more than catalyst speciation and/or activity. In particular, substrate properties (steric and or electronic), purification procedures, or unwanted impurities (facilitating unwanted side reactions) are all reasons that the isolated yields can be lower than expected (compared to the crude mixture). Indeed, it would be more important to relate ppm to TOF to gauge relationships. However, as observed when examining the literature, the majority of papers did not calculate this parameter. In fact, as previously mentioned, TOF should be one parameter calculated and referenced when looking at catalytic reactions to have more understanding of catalytic efficiency. If these values could be obtained, then related Pd concentration (molecular ppm) could provide valuable insights for each catalyst and also each named reaction.

In comparison to the original reported reactions, there are large changes in the concentration of Pd (or transition metal) used in each reaction. Most of the named reactions follow the same trend where the original reported reaction employs a lower in-reaction ppm level of palladium than the average amount for the said reaction. The Kumada–Corriu reaction^78,79^ originally used Ni catalysts at 0.7 and 1 mol % equating to 343 and 833 ppm, respectively. Most of the data follows the original reports with 70% of papers reporting 2 mol % or lower catalyst loadings. The lack of high loadings from this data set causes the average ppm value to be lower than other reactions, i.e., 404 ppm from the 51 reactions examined.

The Negishi reaction^80^ shows a substantial change from the original reporting conditions to those typically used today. In an initial report from 1977, the reaction used 5 mol % catalyst, which we calculated to be 550 ppm in the reaction. As many reactions do tend to use 5 mol %, most (from the gathered data) use significantly less, as low as 0.001 mol %. From the remaining Negishi data, the average result from the 45 reactions analyzed employed an average of 1400 ppm. However, this average is skewed by the wide values through the use of other metals at higher (pre)catalyst loadings.

Similarly, an early report of the Stille reaction^81^ employed a small mole percentage of catalyst compared to substrate, only 0.05 mol % or 110 ppm, clearly lower than the average from 58 reactions found, which was ca. 770 ppm. We noted that 38% of the papers surveyed made use of catalyst loadings below 1 mol % while a notable number (26%) used 5 mol % Pd (pre)catalyst, which is likely to give a higher in-reaction ppm value.

Again, similar to the previously mentioned reactions, the Suzuki–Miyaura cross-coupling reaction^82^ was originally reported to use 1 mol % palladium (pre)catalyst or 179 ppm in the reaction system. This is again like the Stille and Negishi reactions and is lower than the overall average found in the data surveyed. A common occurrence was 1 mol % or lower with 41% of the papers surveyed having a loading below 1 mol %. A total of 75 reactions were found to have an average of 777 ppm.

The relationships previously observed were also revealed for the Sonogashira reaction.^83^ The lowest category of 1 mol % in relation to the substrate gave 167 ppm of Pd (pre)catalyst in the reaction, which was below the average of 869 ppm from the 55 reactions surveyed. The data profile shows a more balanced range of catalyst loadings.

The Heck reaction^84^ was, surprisingly, different from the previous cross-coupling reactions. An early paper from 1972 used 1 mol % catalyst, which was calculated as being over 3000 ppm, well over the average of 800 ppm from 55 Heck reactions. This was due to the lack of solvent used and only three reagents used alongside the (pre)catalyst, i.e., aryl halide, alkene, and base.

Cyanation reactions of organohalides^85^ proved to be interesting; the initial study in 1973 reported the use of both 0.5 and 2 mol %, equating to 551 and 1254 ppm Pd (pre)catalyst, respectively, while he average result was 961 ppm. This was largely due to the use of almost identical moles of Pd(CN)_2_ but with five times less substrate, four times less KCN, and three times less solvent.

A variant of the Buchwald–Hartwig amination reaction (Pd-catalyzed C–N couplings) was reported in 1983 by Migita and co-workers.^64^ A fully expanded methodology, with broad substrate scope, was reported in 1994.^86^ In Migita’s work, 1 mol % of PdCl_2_(*o*-tolyl_3_P)_2_, equating to 1386 ppm, was employed. This high value can be attributed to the solvent constituting only 65% of the total molecular species. In comparison to this, the B–H amination average ppm value is 4800 ppm (vide infra), four times higher than the original report.

## Suzuki–Miyaura Cross-Coupling

The SMCC reaction of organohalides/pseudohalides with organoboron-containing compounds is widely applied with an innate mechanistic complexity that sits beneath its apparent simplicity.^87^ Its wider application enabled easier data extraction in this study. We identified 75 journal articles, which included a total of 97 individual reactions, from which good quality data could be extracted and analyzed.^55,58,59,76,88−137^ The data shows a split between common mol % choices, 0–1 and 4–5 mol %, having ppm values up to 1000 ppm. It also shows that, from these selected papers, lower mol % values are preferred with ca. double the number of 0–1 mol % (40) compared to 4–5 mol % (21). Within the 0–1 mol % tier, the distribution of data is smaller than other mol % tiers, where the data is bunched closer to the lower ppm levels. This could be due to more recent papers being included in the study, where research groups have attempted to reduce catalyst loadings through the use of an activating ligand, i.e., using tailored (pre)catalysts, while maintaining good catalytic reactivity. The lowest mol % used for SMCC reactions was 0.0013, which equates to 0.063 ppm^105^ according to our calculations for molecular ppm. Compared to this, the largest data point observed was 5689 ppm when 7.62 mol % catalyst was used.^133^ Here, the most commonly employed (pre)catalyst is Pd(OAc)_2_ followed by Pd(PPh_3_)_4_, 23% and 22%, respectively, of all reactions, and 15% were variations of PdCl_2_. The mol % and ppm spread (<1, 1–2, 2–3, and 5+ mol % and 8–1500 ppm) across both is quite substantial.

**Figure 5:**
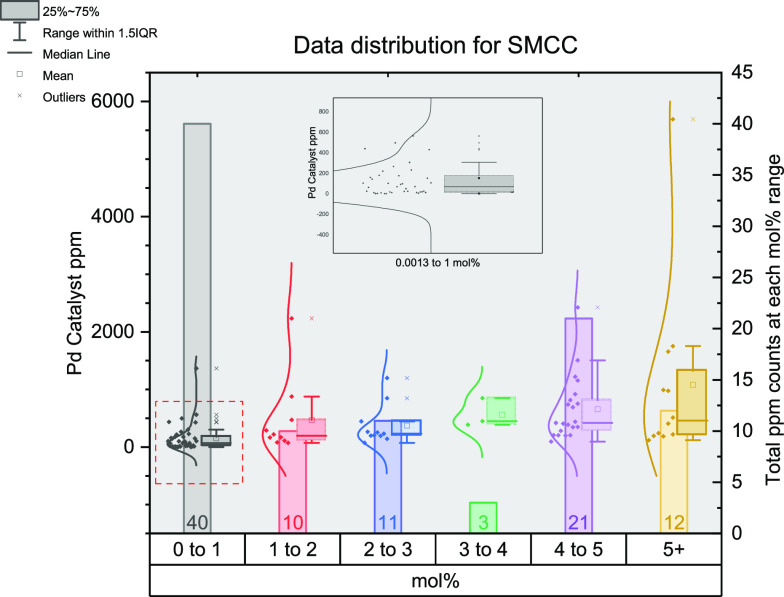
Distribution of data gathered for Suzuki–Miyaura cross-coupling reactions. The *x*-axis shows the mol % of catalysts grouped together (0–1, 1–2, 2–3, 3–4, 4–5, and 5+); the left *y*-axis shows the ppm for each data point; the right *y*-axis shows the total number of data points in each group of mol %. The bar for each mol % is the total data points contained in that mol range. The data points for each mol % group show the distribution of ppm levels while the box plot outlines the range (whiskers), median (internal line), mean (small square), and interquartile range (large box) for each mol % subset.

To represent the large amount of data for each reaction and to help summarize the key trends, a 3-dimensional graph was created (Figure 5). To easily display the data, they have been grouped into mol % categories: 0–1, 1–2, 2–3, 3–4, 4–5, and 5+. Within each of these, the data is displayed in three ways: a bar showing the total number of reactions, a box plot showing the range, middle 50% of data, median, and mean (also included outliers), and then finally, a distribution of all the individual data points. There are many points distributed in the 0–1 mol % category; as such, a zoomed in inset has been included for this category.

The following examples outline the employed ppm levels of Pd used in the published examples of the SMCC reactions. The same format is used for the subsequent reactions, i.e., a summary of key trends/findings followed by relevant examples.

**Scheme 5:**
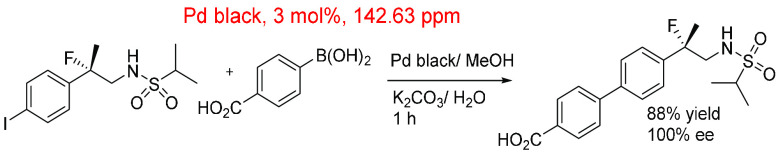
Suzuki–Miyaura Reaction in the Synthesis of LY503430 by Eli Lilly^94^

The development and optimization of LY503430 by Eli Lilly (Scheme 5) made use of a Suzuki–Miyaura reaction to incorporate a biphenyl linkage connected to a sulphonamide containing a quaternary chiral center.^94^ The coupling of the sulphonamide with 4-carboxylphenylboronic acid initially employed Pd(OAc)_2_, PPh_3_, and Na_2_CO_3_ in aqueous IPA refluxing for a total of 3 h. Reaction optimization identified a Pd black catalyst, K_2_CO_3_ base and MeOH as the best reaction conditions. These conditions allowed the reduction in reaction time (to 1 h) and temperature while improving the isolated yield to 88% from ∼70%. Total Pd levels in the purified product ranged from 3 to 8 ppm. Given that this was on a large scale (∼11 L total volume), the in-reaction ppm was low (143 ppm) compared to the higher mol % used (3 mol %). The majority of in-reaction Pd was removed using a Hyflo Super-Cel for filtration and acetic acid for product crystallization, followed by a further filtration.

**Scheme 6:**
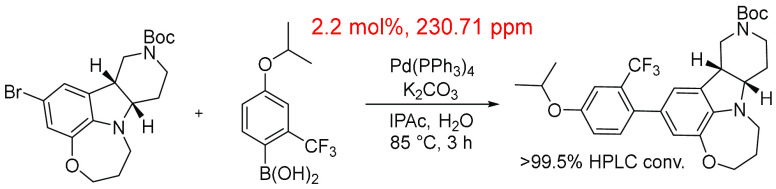
Use of a Suzuki–Miyaura Cross-Coupling Reaction to Synthesize a 5-HT_2c_ Receptor Agonist as Reported by BMS^96^

A large-scale multi-kilogram process to synthesize a 5-HT_2c_ receptor agonist (Scheme 6) was developed by BMS, which gives us an interesting example to assess further.^96^ The product was not formed as a solid and contained high levels of Pd contamination (in the range of 2500–3500 ppm). Further purification of the product was necessary; the use of Picachem carbon or a solution of tris(hydroxymethyl)aminomethane was unable to remove the residual Pd to satisfactory levels. It was only upon a combination treatment using a 20% Na_2_CO_3_ solution of trithiocyanuric acid (<5 °C) where solid Pd precipitate was formed and filtered, and the organic phase was treated using Picachem carbon 80PN, which removed the majority of Pd to give a Pd level of <100 ppm. The “in-reaction” ppm level for the final iteration to form the 5HT_2C_ receptor agonist was 231, which accounts for all the reaction components. The example highlights that the product is very capable of sequestering Pd.

## Kumada–Corriu Cross-Coupling

**Figure 6:**
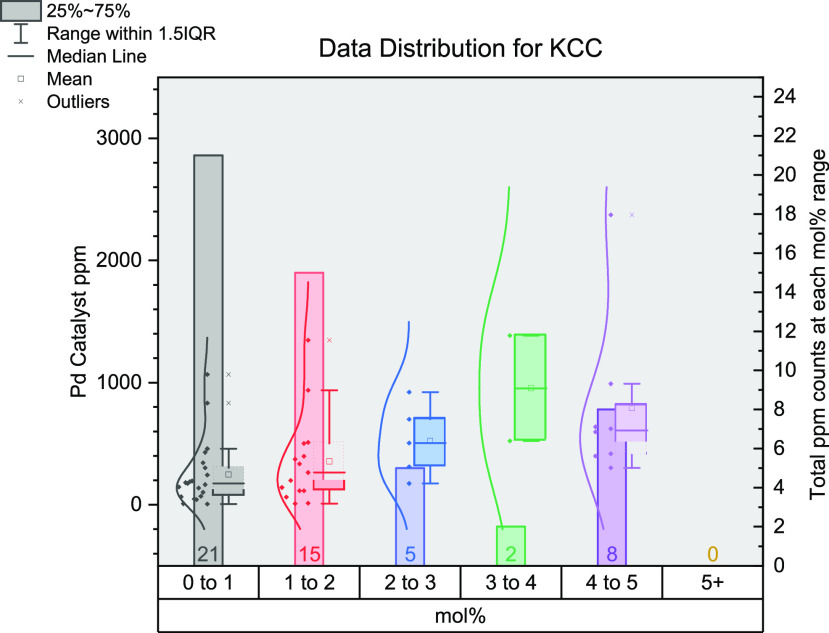
Distribution of data gathered for Kumada cross-coupling reactions. The *x*-axis shows the mol % of catalysts grouped together (0–1, 1–2, 2–3, 3–4, 4–5, and 5+); the left *y*-axis shows the ppm for each data point; the right *y*-axis shows the total number of data points in each group of mol %.

The Kumada–Corriu cross-coupling reaction is well used and understood, being central to many reported multistep syntheses.^138^ From the 43 journal articles (Figure 6) found (comprising 51 reaction sets), the majority make use of lower (pre)catalyst loadings, residing in the 0–1 and 1–2 mol % ranges. It is a rarer occurrence for loadings to be situated in the higher mol % ranges, possibly due to reagent reactivities, sensitivities, or toxicities.^56,78,79,100,112,123,129,138−172^ From the few high mol % values, ppm values reach their maximum from ca. 300–1000 ppm. The lowest mol % value was determined to be 0.1 mol %^161^ (175 ppm, in our survey), which did not give the lowest ppm value. This was found to be from a 2 mol % system calculated at 9.9 ppm (in terms of the Pd (pre)catalyst).^164^ The most commonly employed (pre)catalyst here was Pd_2_(dba)_3_ followed by Pd(OAc)_2_ and Pd(PPh_3_)_4_: 26%, 12%, and 10% of the literature surveyed, respectively.

**Scheme 7:**
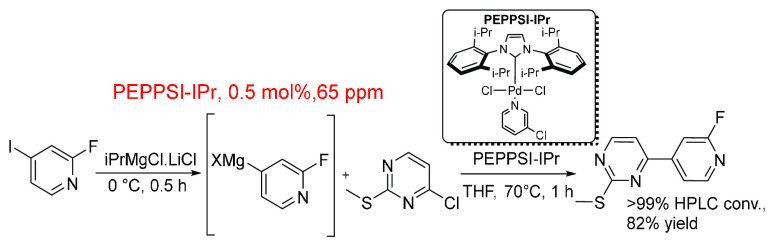
Preparative-Scale Kumada–Corriu Cross-Coupling of Unstable Pyridylmagnesium Halide and Thiopyrimidine^146^

The Kumada–Corriu reaction has also been used in the scale-up of a key intermediate toward an improved synthesis of a pyridine derivative (Scheme 7).^146^ An unstable 4-pyridylmagnesium halide caused scale-up issues. Thus, continuous flow technology (to overcome the scalability issue) and a low temperature Kumada procedure with improved Pd catalysis made the reaction possible. The unstable species was generated by mixing the pyridyl species with a “turbo Grignard” reagent under reaction cooling before being mixed with the heteroaryl species and catalyst at 0.5 mol % (65 ppm). The larger scale reaction was performed using 16 mol of pyridyl halide, 13 mol of thiopyrimidine, 1 mol % PEPPSI-IPr, and 20 L of THF. Even though the mol % of Pd was doubled, there is a considerable difference between the two ppm values (∼7×). This may be due to the larger scale reaction being performed in batch mode and the smaller scale reaction, in continuous flow mode, each having different mixing effects and other physical characteristics and differences.

**Scheme 8:**
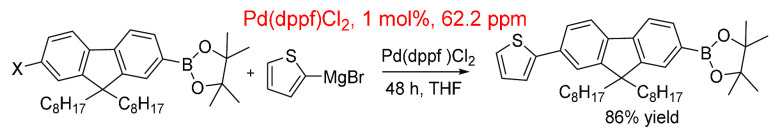
Outline of a Kumada–Corriu Reaction of Halide Fluorenes and Thiopheneyl Magnesium Bromide^167^

Geng and co-workers reported that organoboronic pinacol esters are stable in typical conditions used for the Kumada, Heck, and Stille reactions (Scheme 8).^167^ Fluorene-based organoboron compounds have been used for the preparation of bromo- and iodo-fluorenyl boronic pinacol esters using these cross-coupling reactions. The report outlines each reaction, each with differing conditions. The Kumada coupling employs the lowest catalyst loading (1 mol %), where the reagents have a reaction quantity of ∼0.86 mmol. These and the amount of solvent used, 10 mL, gives an in-reaction ppm level of 62.2 ppm, well below the average from the data gathered. The low ppm level is most likely due to the reaction scale in a large volume of solvent and also provides the highest yield of all three reactions when using the bromo-substituted derivative (86%).

## Stille Cross-Coupling

**Figure 7:**
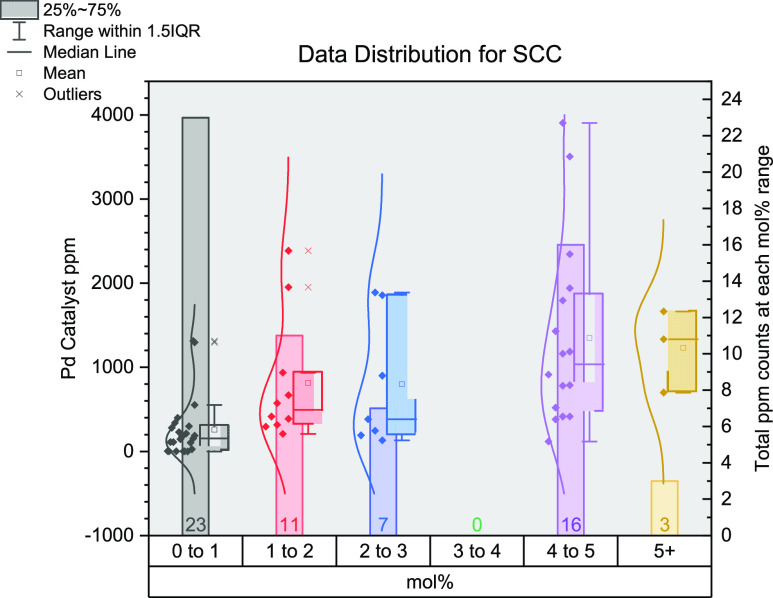
Distribution of data gathered for Stille cross-coupling reactions. The *x*-axis shows the mol % of catalysts grouped together (0–1, 1–2, 2–3, 3–4, 4–5, and 5+); the left *y*-axis shows the ppm for each data point; the right *y*-axis shows the total number of data points in each group of mol %.

The reaction of organohalides/pseudohalides with organostannanes is arguably the most reliable and structurally most resilient cross-coupling reaction.^173^ Unfortunately, the toxicity of organostannyl byproducts is of serious concern. The toxicity of tin reagents is well established, having a toxicity profile similar to that of hydrogen cyanide.^174^ This said analysis of the Stille reaction surveyed here exhibits similar relationships to the Kumada–Corriu reaction. We analyzed 42 journal articles and 61 separate Pd-catalyzed reactions (Figure 7).^27,58,65,81,105−107,166,167,169,171,175−205^ There is a preference for lower mol % ranges, but more reactions are performed at 5 mol %, which is particularly apparent in older articles. There is a larger variation in ppm values for the 4–5 mol % category, most likely due to the large variation in reaction volume. However, this data set has shown the lowest Pd (pre)catalyst loadings for all the named reactions, 0.0001 mol % equating to 0.0082 ppm. Activated substrates can thus employ low levels of Pd catalyst with great effect, where there are limited reaction sensitivities.^65^ The most commonly employed (pre)catalyst here is Pd_2_(dba)_3_ followed by Pd(PPh_3_)_4_: 25%, and 20% respectively.

**Scheme 9:**
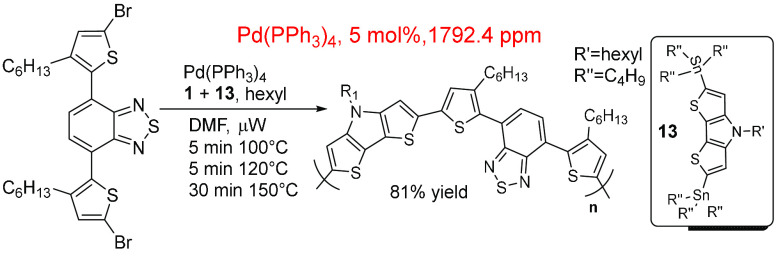
Stille Cross-Coupling Polymerization Reaction Where the Stannate and Halide Are Incorporated into the Same Molecules^,^^201^ Pd(PPh3)4 is used as catalyst at 5 mol % loading.

A recent paper outlines how a stannyl moiety and halide can be incorporated into the same molecule for an effective Stille polymerization cross-coupling reaction (Scheme 9).^201^ This reaction was performed under microwave conditions using 5 mol % Pd(PPh_3_)_4_ (1792 ppm) giving products in ∼85% yield. The in-reaction ppm is high because of the low volume of solvent used and high catalyst loading, compared to the average value.

**Scheme 10:**
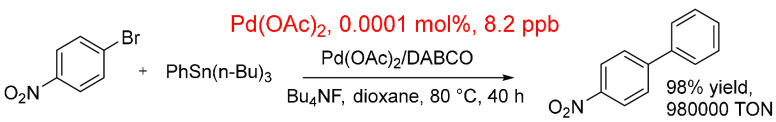
Activated Stille Cross-Coupling Reaction Example Using Ultra-Low Loadings of a Pd Precatalyst^65^

Similarly, an efficient Pd-catalyzed Stille cross-coupling was developed using Pd(OAc)_2_ and DABCO at very low loadings, Scheme 10.^65^ The process enabled access to biaryls, alkenes, and alkynes with catalyst turnover numbers reported up to 980 000. The catalyst loading quoted above represents conditions used for the coupling of 1-bromo-4-nitrobenzene, an arguably nonchallenging substrate (in terms of the weak strength of the C–Br bond). The nature of the reaction conditions (elevated temperature and long reaction time) might indicate a role for small Pd clusters and/or nanoparticles.^25^

## Negishi Cross-Coupling

**Figure 8:**
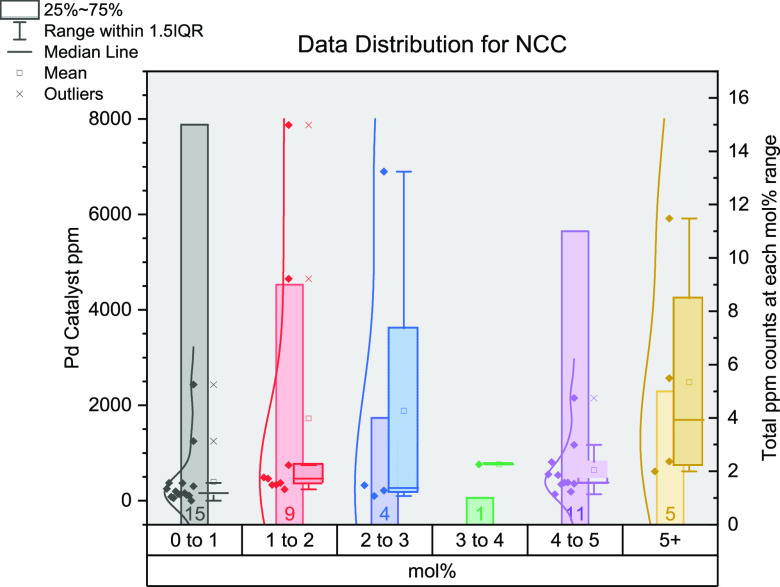
Distribution of data gathered for Negishi cross-coupling reactions. The *x*-axis shows the mol % of catalysts grouped together (0–1, 1–2, 2–3, 3–4, 4–5, and 5+); the left *y*-axis shows the ppm for each data point; the right *y*-axis shows the total number of data points in each group of mol %.

Negishi cross-coupling of organozinc reagents with organohalides is a valuable reaction for a raft of applications, including total synthesis.^206^ The data gathered in this survey spanned 42 journal articles and 45 individual reactions.^57,207−243^ The reaction data shows a preference for lower mol % values, particularly from papers published in the past decade. Interestingly, most ppm data points are distributed to lower ppm levels with more than 60% being below 500 ppm. Only four from the 45 reactions employed no reaction solvent. Out of the remaining 41 reactions, 28 contained solvent in quantities of 90% and above (Figure 8). For the Negishi reaction, there were a wider range of catalysts used.

Five reactions used more than 5 mol % catalyst, and four of them employed transition metals other than Pd, for example, NiCl_2_ at 10 mol % (2566 ppm)^237^ or 20 mol % (616 ppm)^207^ or stoichiometric Ni(cod)_2_ (24 142 ppm).^218^ The only high mol % Pd catalyst employed Pd(dba)_2_ (better referred to as Pd_2_(dba)_3_·dba) was used at 7.14 mol % catalyst loading (822 ppm).^208^

**Scheme 11:**
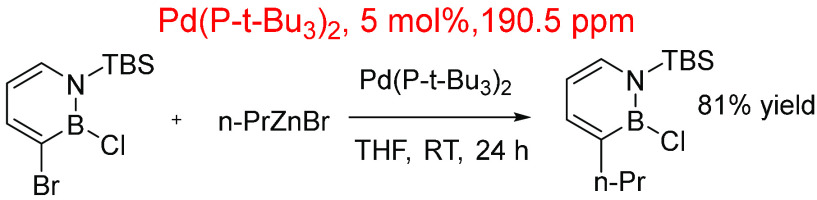
Regioselective Negishi Cross-Coupling Reaction of 1,2-Azaborines Using Pd(P-tBu_3_)_2_ at 5 mol % Loading^210^

Liu and co-workers developed an interesting Negishi cross-coupling protocol enabling the regioselective activation of the C3–Br bond in the presence of the more electrophilic B–Cl bond (Scheme 11). This example shows how a higher catalyst loading (5 mol %) does not follow the general trend as the in-reaction ppm level is 190.5 ppm. For this example, 0.098 mmol of azaborine (0.098 mmol, 33 mM) was used in THF (1 mL, 26 mmol), highlighting the high dilution used for the reaction.^210^

**Scheme 12:**
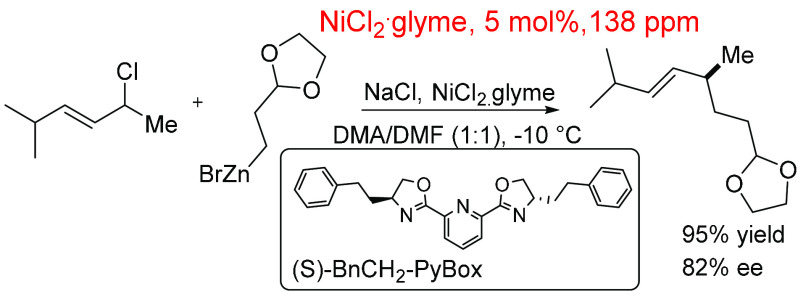
Nickel-Catalyzed Negishi Cross-Coupling Reaction Using NiCl_2_·glyme as (Pre)catalyst at 5 mol %, 138 ppm^229^

Following the previous example, Son and Fu^229^ established an effective Negishi reaction using a NiCl_2_ (pre)catalyst and Pybox (Scheme 12). This reaction employed a higher catalyst loading of 5 mol % but had a low in-reaction (pre)catalyst ppm (138 ppm). It was found that 2-ethyl-1,3-dioxolane zinc bromide provides the highest yields and enantioselectivities for this catalytic system. This system uses a significant amount of solvent (360 mmol) compared to substrate (1 mmol) and catalyst (0.05 mmol), which explains the lower Pd (pre)catalyst ppm value of 138 ppm.

## Sonogashira Cross-Coupling

**Figure 9:**
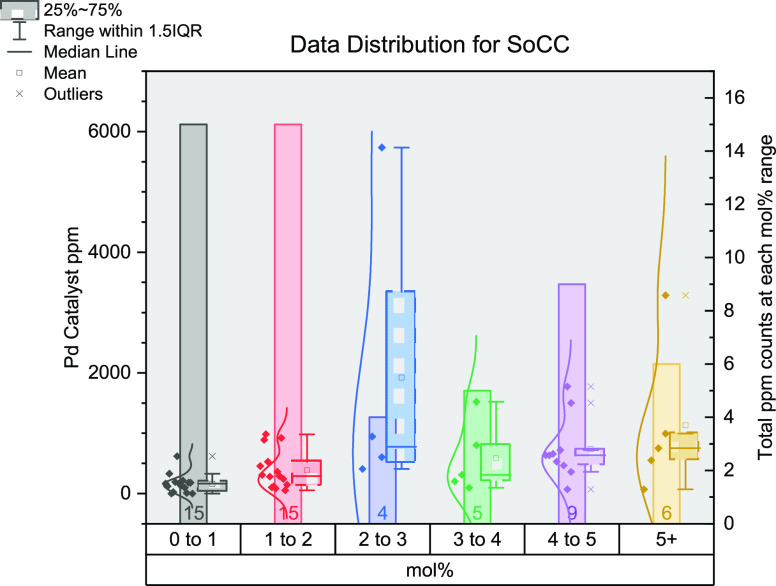
Distribution of data gathered for Sonogashira cross-coupling reactions. The *x*-axis shows the mol % of catalysts grouped together (0–1, 1–2, 2–3, 3–4, 4–5, and 5+); the left *y*-axis shows the ppm for each data point; the right *y*-axis shows the total number of data points in each group of mol %.

The Sonogashira cross-coupling of organohalides with terminal alkynes is catalyzed by Pd and cocatalytic Cu, in the presence of base. It is a valuable reaction for accessing an eclectic array of (typically) internal disubstituted alkynes. Our analysis bares similar trends to Kumada–Corriu and Negishi cross-coupling reactions in that there is a greater use (52% of the data) of lower mol % values. From the journal articles examined (44 articles and 55 reactions, Figure 9),^61,244−276^ almost a third of the reactions make use of 1–2 mol % Pd catalyst with 25% employing 1 mol % or lower. The Sonogashira reaction shows a trend toward lower ppm values, typically in the 500 ppm and lower region (median value of 360 ppm). There are also higher ppm values that skew the average to 883 ppm. The highest mol % category contains only 10 mol % reactions where the ppm values range from 550^120^ to 13 000^270^ ppm. The lowest ppm was calculated to be 0.17 from a reaction using 0.001 mol % Pd catalyst loading.^260^ Approximately 25% of all (pre)catalysts used were PdCl_2_(PPh_3_)_2_ with the remaining 75% being spread over all other (pre)catalysts, although variations of (with different ligands) PdCl_2_ were the most common making up 45% of the total.

**Scheme 13:**
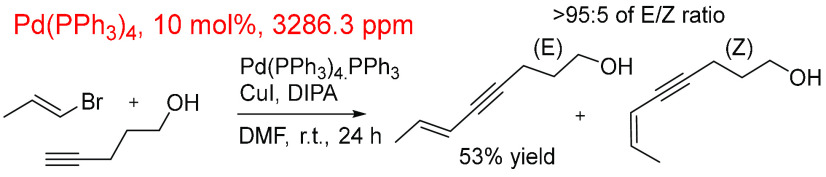
Sonogashira Cross-Coupling or an Alkyl Bromide with Alkyne Using Pd(PPh_3_)_4_, PPh_3_, and CuI^,^^252^ Pd catalyst loading was 10 mol % at 3350 ppm Pd.

Following this, Pastre and co-workers^252^ described a methodology in the synthesis of pharmaceutically relevant 1,3-enyne derivatives (Scheme 13). Stereoselectivity was an integral part of the study about the Sonogashira reaction where the (*E*) and (*Z*) products were obtained at 81% and 68% yields, respectively, from the corresponding *E*- and *Z*-vinyl bromides. The reaction made use of a high Pd catalyst loading (10 mol %, 3350 ppm), significantly higher than most other examples, which sit closer to the average (883 ppm). Here, solvent contributes just over 80% of the reaction total (0.36 mol) compared to the substrate (0.013 mol).

**Scheme 14:**
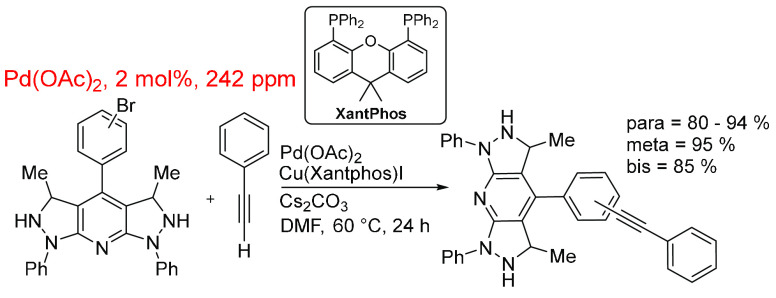
Sonogashira Cross-Coupling Reaction Catalyzed by Pd(OAc)_2_ (2 mol %, 242 ppm) Alongside Cu(Xantphos)I and Cs_2_CO_3_ in DMF^269^

Yin and co-workers^269^ devised a methodology to synthesize bis-pyrazolo pyridine derivatives with the final step being a Sonogashira reaction to introduce the alkynyl moiety (Scheme 14). The Sonogashira reaction here is not the focus of the report but adds the possibility of cross-coupling reactions to bispyrazolo pyridines. This reaction system employed Pd(OAc)_2_ at 2 mol % catalyst loading along with CuI(Xantphos) and Cs_2_CO_3_ as the base. The Pd (pre)catalyst has an in-reaction value of 242 ppm, representing another example of solvent being the major reaction component.

## Heck Alkenylation Cross-Coupling

**Figure 10:**
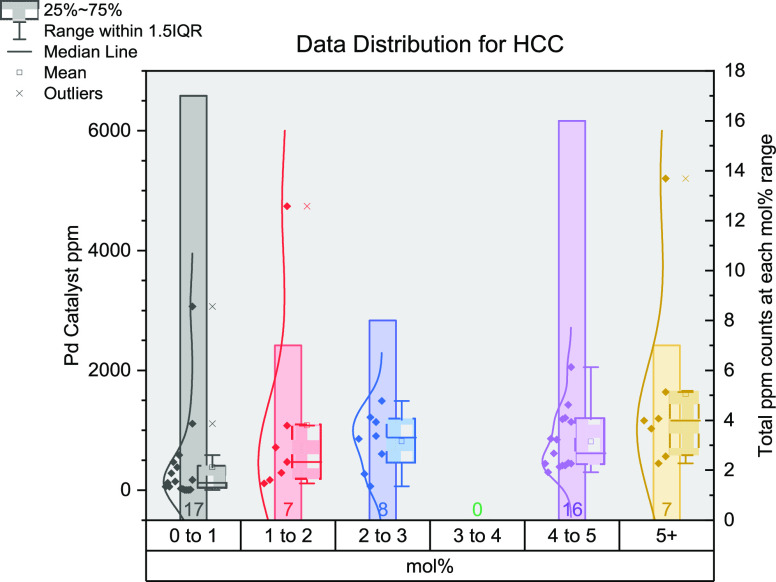
Distribution of data gathered for Heck cross-coupling reactions. The *x*-axis shows the mol % of catalysts grouped together (0–1, 1–2, 2–3, 3–4, 4–5, and 5+); the left *y*-axis shows the ppm for each data point; the right *y*-axis shows the total number of data points in each group of mol %.

The Heck reaction of an alkene with an organohalide historically represents one of the oldest Pd-catalyzed transformations. Our literature search for appropriate Heck reactions involved the identification of 45 articles, which detailed 55 reactions (Figure 10).^60,115,277−309^ The distribution of mol % values are similar to those seen in the Stille cross-couplings. There are equal numbers of 0–1 and 4–5 mol % although the ppm distribution is wider, ranging from 3.3 to ca. 2500 ppm. The lowest mol % category gives the lowest ppm value (3.3 ppm).^115^ There is slight variation in the lower mol % category where, for example, 1 mol % gives 10.7 ppm but conversely a 0.5 mol % reaction gives 1105 ppm. This is largely due to the 0.5 mol % reaction being run neat (no solvent). Here, 32% of the (pre)catalysts employed were Pd(OAc)_2_; 22% were variations of PdCl_2_.

**Scheme 15:**
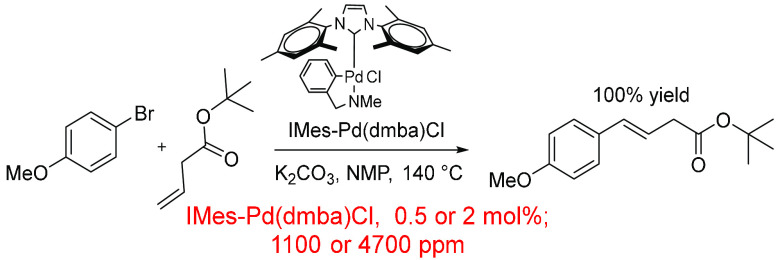
Heck Cross-Coupling of Functionalized Aryl and Heteroaryl Bromides by IMes-Pd(dmba)Cl^283^

Another example that shows the role of solvent in dictating the in-reaction ppm was uncovered in the development of a highly active NHC-Pd (pre)catalyst.^283^ This (pre)catalyst was used to couple an aryl bromide and an alkene in a Heck reaction (Scheme 15). The schematics in the paper report the use of NMP as the reaction solvent, run at 140 °C for 18 h. However, the supporting information from the original publication did not report the amount of NMP used in the general Heck procedure. This means that the in-reaction ppm calculation may be higher than that used in practice. The calculated ppm values were ca. 1100 ppm at 0.5 mol % catalyst loading and ca. 4700 ppm at 2 mol % catalyst loading. The use of 1 mL of NMP would dramatically reduce the relative amount of Pd (pre)catalyst in the reaction, e.g., 0.5 mol % = 334 ppm and 2 mol % = 1300 ppm.

**Scheme 16:**
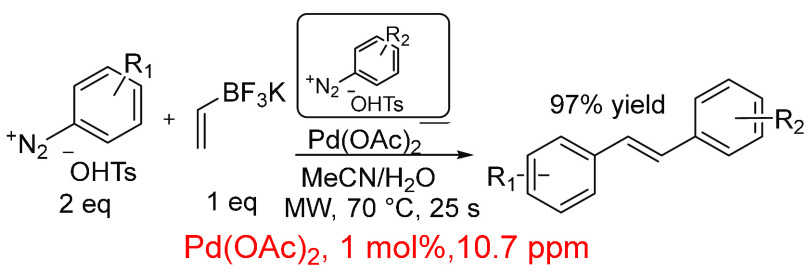
Preparation of Styrenes and Vinylheterocycles Using a Heck Cross-Coupling Reaction^290^

Reactive arenediazonium salts can be used in Heck-type couplings (often referred to as Heck–Matsuda reactions^303^) and were used to prepare sensitive styrenes, which serve as substrates for a range of symmetrical and unsymmetrical stilbenes, formed by a sequential workflow synthesis of Suzuki–Miyaura and Heck reactions (Scheme 16).^290^ Reactions were performed at low palladium loadings (1 mol %, 10.7 ppm) and required a short amount of time for the reaction to be completed (several minutes), and turnover frequencies were high, typically >2000 h^–1^.

## Cyanation of Organohalides

**Figure 11:**
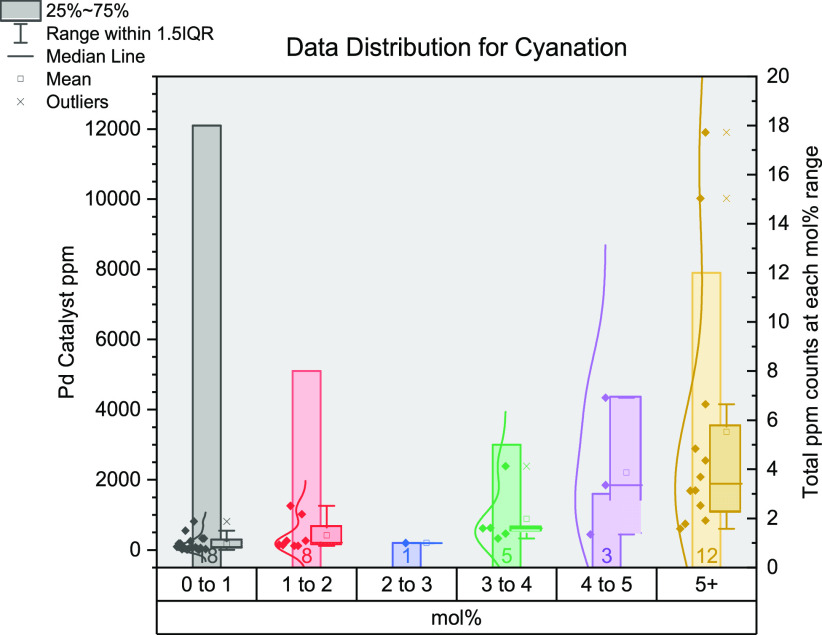
Distribution of data gathered for catalytic cyanation cross-coupling reactions. The *x*-axis shows the mol % of catalysts grouped together (0–1, 1–2, 2–3, 3–4, 4–5, and 5+); the left *y*-axis shows the ppm for each data point; the right *y*-axis shows the total number of data points in each group of mol %.

Organohalides react with a variety of cyanating agents in the presence of a metal catalyst (typically) to afford organocyanide products, which are useful precursors to a range of products, including amines^310,311^ and carbonylic acids.^312,313^ Our literature survey identified 40 suitable journal articles with 47 reactions described (Figure 11).^62,85,121,124,282,300,314−346^ Copper,^314,317,327,333,338,344^ nickel,^322,340^ and ytterbium^344^ make up most of the 5+ mol % category where catalyst loadings are 10 mol % (743 ppm lowest value) or above with the highest value of 130 mol % (11 900 ppm). There is a greater variation in the mol % values used with many more found in the 3–4 mol % category. Greater than 50% of the data is above 1 mol % and has a maximum catalyst ppm of 11 900 (130 mol %^314^). The greatest range is seen in the >5 mol % category for both mol % and ppm: 7.5–130 mol % and 606.2–11902 ppm, respectively.^314,320^ For this reaction, 13% of all entries employed Pd_2_dba_3_ and 13% used Pd(OAc)_2_ as (pre)catalysts. Many entries used various forms of Pd^0^/PdNPs (15%).

**Scheme 17:**
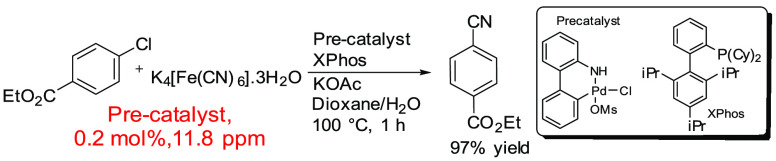
Catalytic Cyanation of Aryl Chlorides Using 0.2 mol % of (Pre)catalyst, 11.8 ppm^316^

There is evidence for the improvement of catalytic cyanation reactions in more recent papers. Buchwald outlined that catalytic cyanation reactions can occur using a nontoxic cyanide source (K_4_[Fe(CN)_6_]·3H_2_O) while still maintaining catalytic efficacy (Scheme 17).^316^ The use of a palladacycle (pre)catalyst and ligand (XPhos) using a low loading (0.2 mol %) gives a total in-reaction Pd ppm of 11.8 ppm. A total of 5 mL of solvent (dioxane/H_2_O; 1:1) was used, which may explain why Pd ppm levels were low. Low (pre)catalyst loadings enabled high yields to be achieved, up to 90%.

**Scheme 18:**
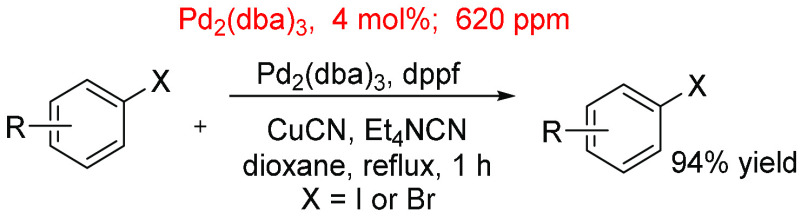
Pd-Catalyzed Cyanation of Aryl Halides Using Pd_2_dba_3_ at 4 mol %, 620 ppm^315^

Conversely, higher loadings and in-reaction ppm levels were shown to be effective when used for the catalytic cyanation of aryl halides employing CuCN/Et_4_NCN as the cyanide source (Scheme 18).^316^ The Pd catalyst and ligand combination {Pd_2_(dba)_3_ and dppf ligand, likely generating Pd(η^2^-dba)(dppf) in situ^347^} generally shows good to high yields after each reaction, employing catalyst loadings of 4 mol % (∼620 ppm). The work demonstrates that a simple Pd(0) precursor and ligand (dppf) can function well in this type of chemistry.

**Scheme 19:**
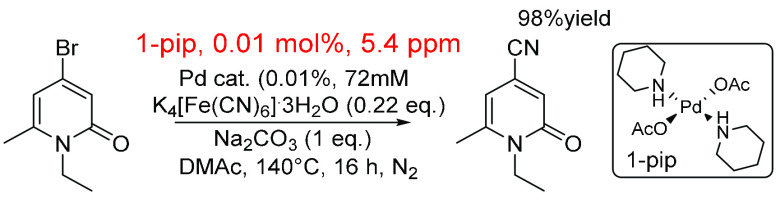
Catalytic Cyanation Reaction Using 1-pip as Catalyst, 0.01 mol % and 4.9 ppm^326^

The cyanation of a *N*-benzyl 4-bromo-pyridin-2-one derivative has been shown to occur using a Pd (pre)catalyst consisting of *trans*-piperidine and *trans*-acetate ligand (1-pip). The (pre)catalyst in this system was used at a very low loading (0.01 mol %), which equates to 4.9 or 7 ppm in terms of mass (Scheme 19).^326^

During this study, several catalysts were synthesized, and their efficacy was tested during the arylcyanation of aryl bromides. It was noted that water (derived from K_4_[Fe(CN)_6_]·3H_2_O) played a critical role in the mechanism, which was switched from being homogeneous to heterogeneous. Even when using such low loadings, the catalyst showed good activity and converted 50% of the substrate to product. Kinetic studies showed a change in TOF and TON when using K_4_[Fe(CN)_6_] (<220 ppm of H_2_O) versus K_4_[Fe(CN)_6_]·3H_2_O (>4000 ppm of H_2_O) where higher TON and TOF were found with the anhydrous cyanating agent.

## Buchwald–Hartwig Amination

**Figure 12:**
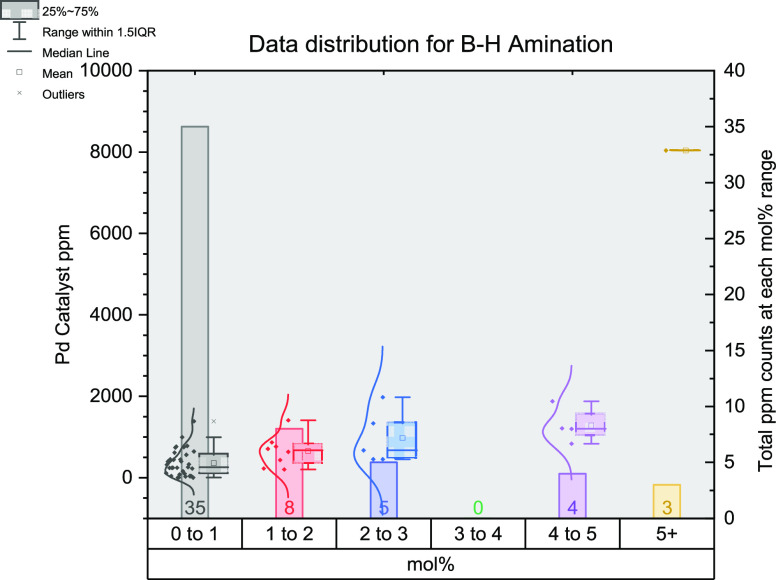
Distribution of data gathered for Buchwald–Hartwig amination reactions. The *x*-axis shows the mol % of catalysts grouped together (0–1, 1–2, 2–3, 3–4, 4–5, and 5+); the left *y*-axis shows the ppm for each data point; the right *y*-axis shows the total number of data points in each group of mol %.

The Buchwald–Hartwig amination reaction of organohalides with organoamines affords an eclectic array of new products containing new carbon–nitrogen bonds. Data extraction of appropriate Buchwald–Hartwig amination reactions led to a similar distribution of articles to the SMCC data set (Figure 12). From 57 articles and 69 reactions,^64,86,125,126,348−400^ the largest proportion, again, are localized in the lowest mol % category of 0–1 mol %. There are several sources that involve the use of other metal additives (CuI and Zn(OTf)_2_), which skew the data, being used in 50 (0.5 equiv.) and 150 mol % (1.5 equiv.) respectively. Disregarding these two non-palladium species, the highest Pd value is 8.33 mol % (8041 ppm) while the lowest is 1.34 ppm, with a global average of 1885 ppm. The same general trend is followed where a higher mol % means a higher in-reaction ppm, but there remains a large variation in the 0–1 mol % category where the higher values are close to those found in the 4–5 mol % category. The most common (pre)catalysts were specialist catalysts containing bulky ligands, e.g., PEPPSI (23%), followed by Pd_2_dba_3_ (28%) and Pd(OAc)_2_ (22%) with various ligands.

**Scheme 20:**
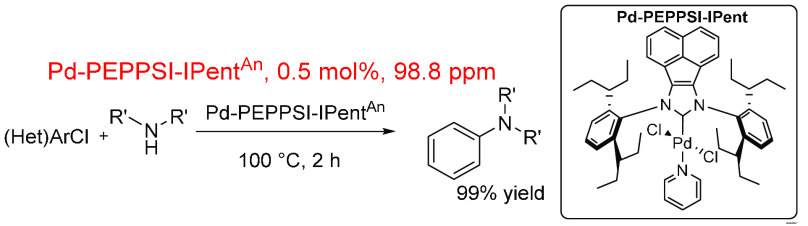
Palladium (Pd-PEPPSI-IPENT^An^) Catalyst Buchwald–Hartwig Amination of Deactivated Aryl Chloride under Aerobic Conditions^379^

An NHC-containing, palladium (pre)catalyst was used to catalyze a Buchwald–Hartwig amination reaction of deactivated heteroaryl chlorides (Scheme 20).^379^ A low mol % loading of (pre)catalyst was used, which gave a low in-reaction ppm of 98.8, well below the average value for this reaction set (4820 ppm). However, this average considers the large ppm values from other metal sources; when these are removed, the average is reduced to 818 ppm. Such a low ppm value for this reaction may be indicative of a more active catalytic species derived from the initial (pre)catalyst.

**Scheme 21:**
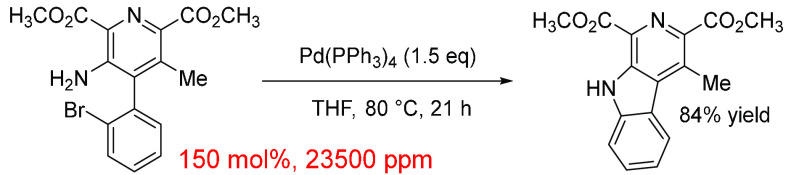
Use of an Intramolecular Buchwald–hartwig Amination Reaction in the Total Synthesis of Lavendamycin^381^

An intramolecular Buchwald–Hartwig amination reaction (Scheme 21)^381^ has been used in the total synthesis of lavendamycin, a pharmaceutically relevant natural product. Pd(PPh_3_)_4_ was used as the catalyst in a very high molar ratio (150 mol %, 1.5 equiv), which gives an overall contribution of 23 500 ppm to the reaction. This is an incredibly high Pd loading and may be explained by (other than the high molar equivalents) the lack of additional reagents within the reaction with only solvent (THF) playing a role in the ppm calculation, 96% of the total molecular amount.

## Direct Arylation

**Figure 13:**
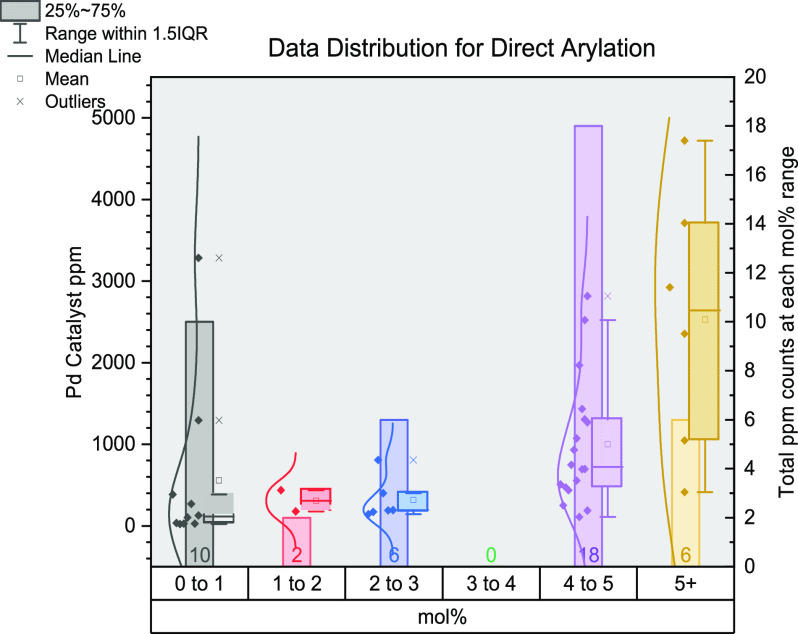
Distribution of data gathered for direct arylation cross-coupling reactions. The *x*-axis shows the mol % of catalysts grouped together (0–1, 1–2, 2–3, 3–4, 4–5, and 5+); the left *y*-axis shows the ppm for each data point; the right *y*-axis shows the total number of data points in each group of mol %.

The reaction of (hetero)aryl halides with appropriate organic substrates containing reactive C–H bonds has emerged as a competitive and complementary reaction to SMCC reactions. We identified 40 journal articles for direct arylation with a total of 42 reactions, which were appropriate for data extraction (Figure 13).^63,328,401−436^ 18 of these make use of 5 mol % (pre)catalyst (42% of the total for this set), and 24% contain the 0–1 mol % category; the remaining reactions make up the rest (32%). There is a large variation in the ppm levels in the 5 mol % category from the lowest value of 109 to 2800 ppm, and the an average is 998 ppm. These values are considerably higher than the other, lower mol % categories, which have an average of 449 ppm. Conversely, the highest ppm value calculated was contained in the 0–1 mol % category with a catalyst loading of 0.5 mol %. This high value seems to be due to the other reagents contributing much less to the reaction mixture (0.1, 0.15, 0.05, 0.005, and 0.03 mmol) while still having good reaction yields above 50%. Here, 83% of catalyst species were Pd(OAc)_2_ spanning the full range of ppm values and all mol % categories.

**Scheme 22:**
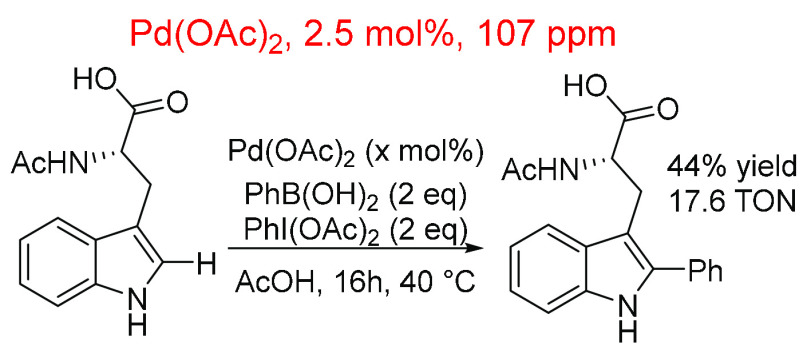
Arylation of an N-Acetyl Protected Tryptophan Using Pd(OAc)_2_ as Catalyst and a Variety of Loadings (1, 2.5, 5, and 10 mol %)^,^^412^ 2.5 mol % = 106.88 ppm.

Fairlamb and co-workers mentioned, explicitly, the need to consider ppm Pd in direct arylations in a study that showed that Pd nanoparticles were formed as highly effective catalyst species for direct arylation reactions in situ from simple Pd (pre)catalysts in the presence of polar aprotic solvents (e.g., DMF).^412^ The work highlighted the correlation between Pd concentration (ppm) and catalyst TON showing that contrary to the expectation (in a rate = *k*[cat][A][B] regime under which TON should be measured) a lower Pd concentration gives a higher TON; this is the opposite of assuming [Pd] = [cat]. Using Pd(OAc)_2_ (2.5 mol %) in the C2-arylation of tryptophan (Scheme 22) gave a catalyst concentration of 50 ppm and a TON of 17.6 in comparison to using 10 mol % (200 ppm) and a TON of 6.6. Pd nanoparticles (PdNPs) seem to play a major role in this reaction as their formation was observed within 15 s of (pre)catalyst addition.

**Figure 14:**
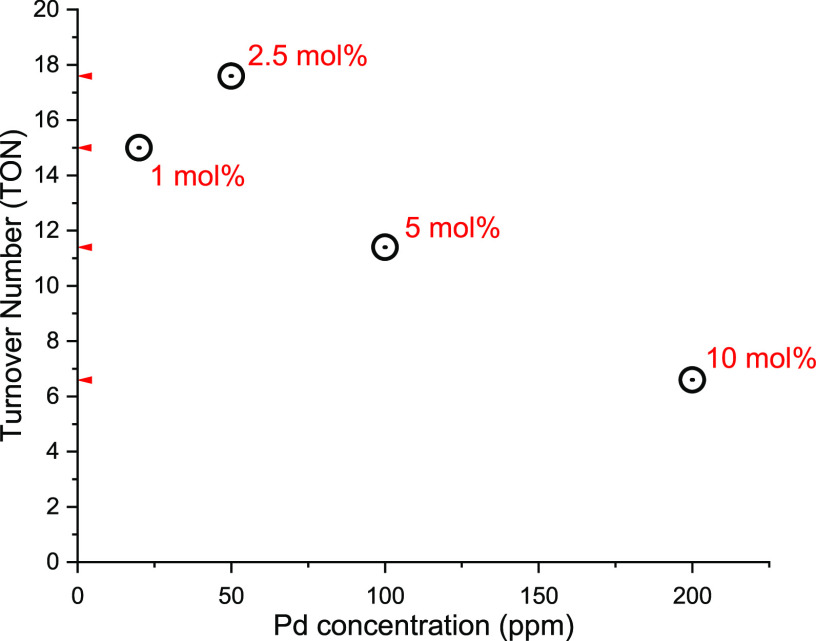
Relationship between catalyst TON and Pd (ppm) concentration shows a general negative trend of the reaction in Scheme 22.^412^

The relationship between Pd concentration (ppm) and TON, particularly in this example, shows a general negative trend: as TON increases, the Pd concentration (ppm) decreases (Figure 14).

**Scheme 23:**
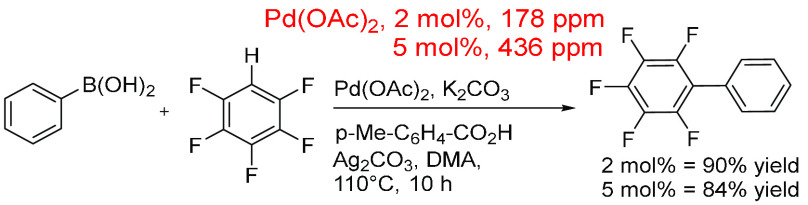
Direct Arylation of Electron Deficient Pentafluoroarenes with Arylboronic Acids through an Oxidative Process Using Pd(OAc)_2_ Either at 2 or 5 mol % (178 or 436 ppm)^425^

A method for the direct arylation of pentafluoroarenes with arylboronic acids (Scheme 23) was shown to have a broad substrate scope for both arenes and boronic acids. This was highly dependent on the acidity of polyfluorobenzene, which determined which base was used.^425^ A silver salt was also used as a reaction additive at 2 equiv of substrate (0.2 mmol), which will play a decisive role in the reaction mechanism. The low Pd ppm here (2 mol % = 178 ppm; 5 mol % = 436 ppm) could be, again, attributed to the amount of solvent (DMA, 2 mL, 0.021 mol), a factor of a hundred higher than the polyfluoroarene substrate.

**Scheme 24:**
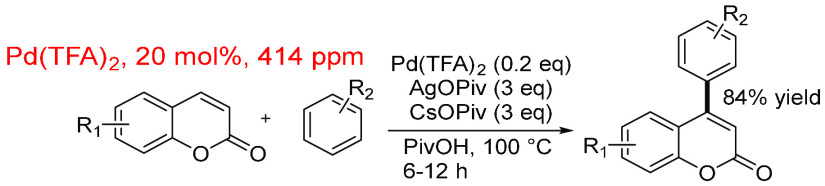
Direct C4-Arylation of Various Coumarins^,^^437^ A variety of R1 and R2 groups were used in different positions around each aromatic ring.

The next example from the direct arylation data set (Scheme 24) indicates how a high mol % of (pre)catalyst can still provide a modest in-reaction ppm, i.e., 20 mol % and 414 ppm. The reaction of coumarin derivatives with substituted arenes, using Pd(TFA)_2_ in pivalic acid along with silver and cesium pivalate salts, typically shows good yields (41–84%). The other reagents (arene, silver pivalate, cesium pivalate, and pivalic acid) are in high molar quantities in this reaction system compared to the coumarin reagent. The in-reaction ppm (414 ppm) for this system is well below the total direct arylation average, having a value of 941 ppm.

## Further Analysis of Selected Articles Reported in the *Journal of Medicinal Chemistry*

As a comparison to the previous data set, ca. 15 additional papers were surveyed from the *Journal of Medicinal Chemistry* (*JMC*) for both SMCC reactions^133,204,438−453^ and Buchwald–Hartwig amination reactions.^383−388,390−396^ As the previous data were found from a variety of journals, although largely catalysis specific, we were interested to see how different these would be compared to papers only reported in *JMC* and if there was a difference in mol % and ppm catalyst levels. Finding more papers with a greater focus on catalyst usage, rather than catalyst optimization, reveals a different angle to this study. In addition, we examined the change catalyst ID from two selected years over the course of multiple issues. We took a small random selection, evaluating issues 1 to 11 from 1985 in *JMC*, which revealed some interesting results. Pd/C was a common catalyst, often used when performing hydrogenations, with 91 entries for Pd/C versus three other Pd catalysts. When issues 1–6 from 2017 in *JMC* were studied, a large proportion employed Pd/C (45%, 52 papers) compared to other Pd catalysts, e.g., Pd_2_(dba)_3_, Pd(OAc),_2_ or Pd(PPh_3_)_4_ (55%, 63 papers).

Using the data from the *JMC* SMCC papers, despite being targeted and arguably limited, highlights the popular catalyst choice as well as the employed ppm Pd in more applied systems. The trends show the skew toward higher mol % values with 45% occurring in the highest mol % range or 68% of the reactions performed at 5 mol % loading or above. Again, all reactions in the 4–5 mol % category use 5 mol % alone. In contrast to the other data from the SMCC reaction data, there are no data points in the lowest mol % category (0–1 mol %), where the majority were found previously (40 reactions). These *JMC* reported reactions largely use either PdCl_2_(dppf) (27%) or Pd(PPh_3_)_4_ (41%) compared to the original data set where Pd(OAc)_2_ (29%) and Pd(PPh_3_)_4_ (16%) were found to be the most common.

**Figure 15:**
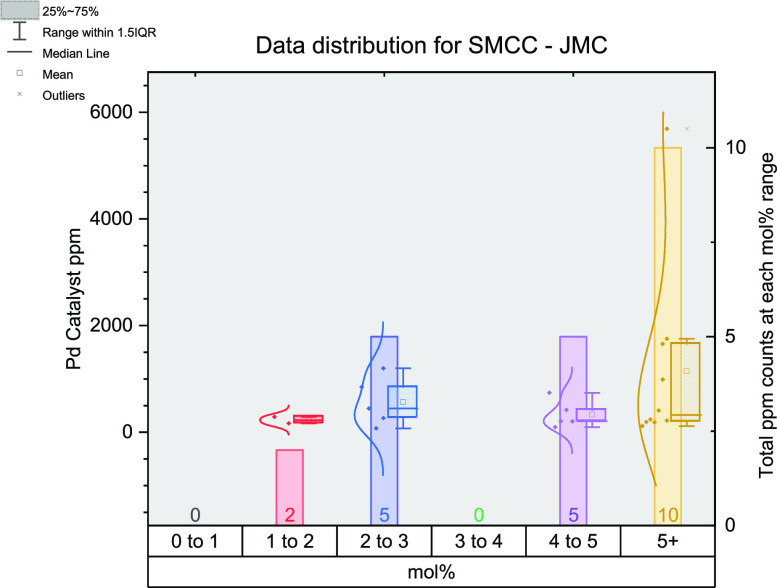
Figure outlining the data extracted from the *Journal or Medicinal Chemistry* for SMCC reactions.

**Figure 16:**
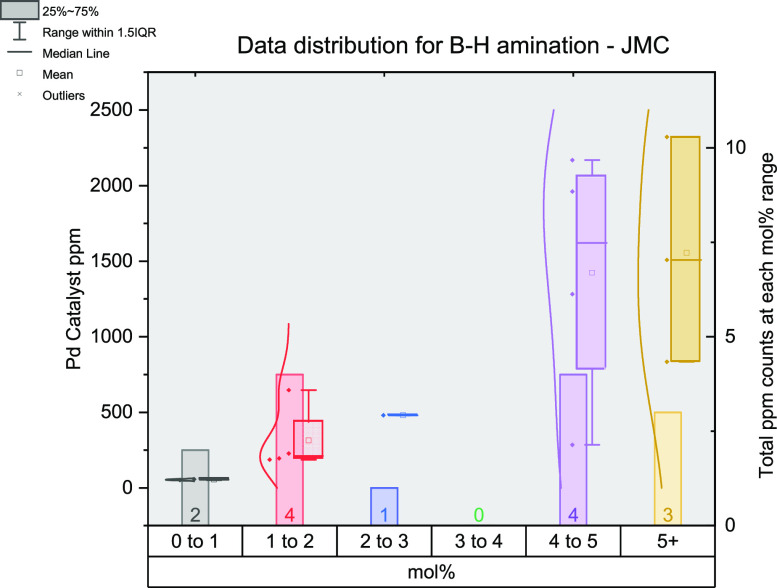
Figure outlining the data extracted from the *Journal or Medicinal Chemistry* for Buchwald–Hartwig Amination reactions.

There is a large change in the number of reactions in the 0–1 mol % range for both Buchwald–Hartwig amination and SMCC reactions. For the latter, there are 40/76 in the original set versus 0/22 in the *JMC* set, and for the former, there are 35/57 in the original set versus 2/14 (Figures 15 and 16) in the *JMC* set. The identity of the (pre)catalyst used changes between the original set and *JMC* set: Pd_2_(dba)_3_ (45%) or Pd(OAc)_2_ (14%) being the most often used versus 16% and 22% for the same catalysts.

## Conclusion

This study has revealed that there is a variation in Pd catalyst usage and quantities described across a range of cross-coupling reactions. Particularly apparent is how experimental data is reported with no standardized format, a topic that has been discussed widely elsewhere, but a general format has yet to be agreed upon. Our metric, molecular ppm, has given us a valuable and fair insight and an easy method to calculate Pd ppm to assist in a comparison of the concentration of Pd in a large variety of reported cross-coupling reactions. This metric was chosen so that a clear method of comparing Pd concentration across many papers in the literature could be pragmatically established, where there were significant differences in the reporting of reaction data. In general, while considerable manual data processing was necessary (in our study), most data does conform to a good standard of experimental and academic knowledge. It was evident that the data reporting has improved greatly, particularly over the last 15 years or so where it has been more common to see detailed procedures reported. Reported data should include all that is necessary for the work to be repeated independently, and sometimes; In some cases we noticed that key sections of information were omitted. Particularly now, in such a data rich era, where most chemical reaction information is electronic, it should be easier for all essential data to be included whether directly in the report or, as happens more often, included in the associated paper supporting information. Giving thought to how one’s data can be harvested by appropriate program scripts and computational methodologies will be important moving forward.

If one variable in a study is claimed to be novel (or a potential selling point of a particular study), then we recommend that further calculations support the claims made. The calculation of the Pd ppm concentration of the stock solution is the minimum as the concentration of Pd will immediately change when added into the reaction medium (including all reagents and solvents). Arguably, of equal importance is knowing how long a Pd catalyst stock solution has been standing prior to deployment (an issue for high throughput screening campaigns particularly). This has been shown to be an issue before and could contribute to formation of unknown products and uncontrolled side reactions. We note that the use of “ppm level” palladium in catalytic reactions is not novel and can be observed in a large proportion of the papers we have studied. Indeed, we make the point that one should examine it and report it for all Pd-catalyzed cross-coupling reactions.

**Figure 17:**
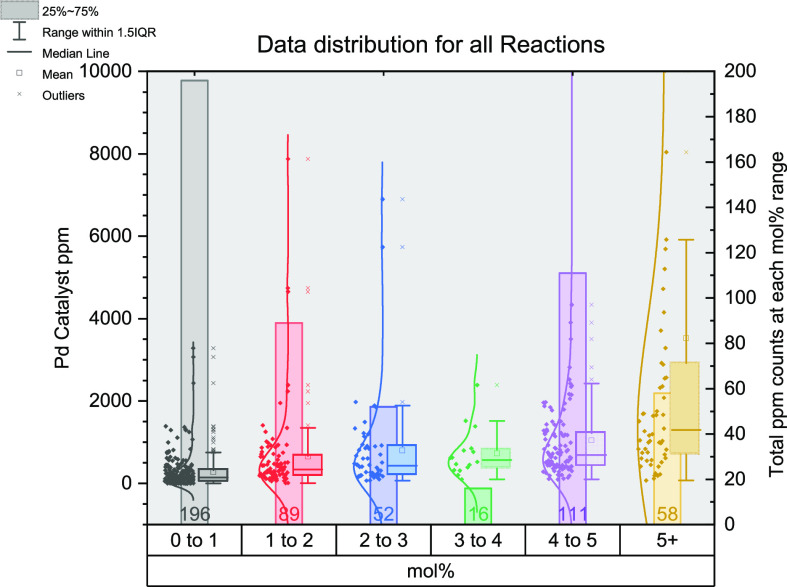
Figure outlining all data gathered throughout this study.

Regarding the ppm data from each cross-coupling reaction (Figure 17), there is a general trend of in-reaction ppm levels increasing as the catalyst loading increases. However, the ppm levels are largely dependent on the number of other species used in each reaction and the volume of solvent. Solvent volume plays a large role in dictating the overall in-reaction ppm as this is often >90% of the mixture. Understanding the amount of Pd or indeed any (pre)catalytic species present in the reactions may help to understand the level of active catalytic Pd and what levels of (pre)catalyst are needed. The amount of catalyst present is not the only factor to be considered as important (TON, TOF, and catalyst deactivation triggers are important parameters to know) but may present more depth in the understanding of catalytic reactions compared to only using moles or catalyst to substrate ratios (mol % values). Solvent polarity can often have significant effects on the reaction yield as well as changing the ratio of phosphine ligand to Pd (pre)catalyst.^24,454−456^ The choice of Pd (pre)catalyst may also change the reactivity with different substrates. These reactions, and indeed each Pd catalyzed system, are complex with many factors affecting the overall reaction outcome. The use of low Pd loadings (low molecular ppm values) could be due to the cost of the (pre)catalyst or cost of ligand used alongside the Pd (pre)catalyst. Clearly, this needs to be examined critically in an independent study. Many phosphine ligands tend to be expensive, which could directly affect the employed (pre)catalyst loading. Lower loadings may also be employed when using more specialized (pre)catalyst species. There are a variety of specialist (pre)catalysts available for cross-coupling reactions. That being said, popular choices still remain, e.g., Pd(OAc)_2_, Pd_2_(dba)_3_, Pd(PPh_3_)_4_, and Pd(dppf)Cl_2_.

The level of Pd in reactions (particularly in academic studies) is usually given as a ratio of the limiting reagent. Keeping in mind the amount of Pd at the beginning, during, and after the reaction could lead to valuable knowledge pertaining to the state of Pd and if certain products sequester it. A full palladium balance (or other metal catalyst) could be useful in the full optimization of the Pd-catalyzed systems. The improvement of the sustainability and Pd catalyst recovery is an important factor for sustainable Pd-catalyzed cross-couplings. This is already regulated in the pharmaceutical industry requiring 10 μg g^–1^ as an oral concentration of the active pharmaceutical ingredient (API) as a general guideline, which is primarily determined by dose levels.^457^

Ultimately, from the entire data set, we recommend the following points are considered. Where possible, calculate Pd ppm in the reaction considering all components (in either mg kg^–1^ or molecular ppm) and state all details of the experimental properties, including quantities (g, mmol, M, mL, and catalyst ppm), solvent, and additives. Journals do have different but specific guidelines to follow regarding the location of experimental details, whether that be in the text (below tables, figures, or schemes), in the article but within an experimental section, or within the associated supporting information. Additional data could also be useful to determine the trends or mechanistic details if the Pd concentrations are provided alongside the reaction outcomes, i.e., TON, TOF, product yield, and conversion.

**Scheme 25:**
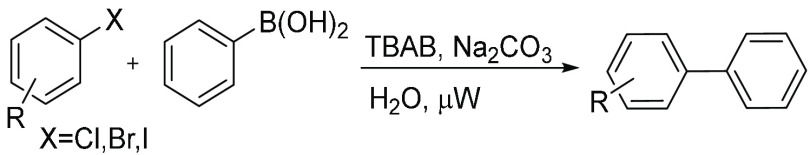
SMCC Reaction of Aryl Halides and Aryl Boronic Acids in Water

Lastly, there have been several studies reporting “Pd-free” catalytic cross-coupling reactions, particularly for the SMCC reaction. Leadbeater and Marco’s^458^ thorough reassessment of the transition metal-free SMCC reaction showed that trace sub-ppm Pd concentrations, Scheme 25 (found in a metal carbonate base), can effectively mediate the reactions of activated aryl halide substrates, e.g., 4-bromoacetophenone, at high temperatures using microwave irradiation or conventional heating, the latter less effectively.

The issue of trace Pd being carried through an amine ligand synthesis has been highlighted,^459−461^ leading to the retraction of a high-profile paper describing an amine-catalyzed SMCC reaction of aryl halides and arylboronic acids.^462^ The consideration of the ppm levels of Pd in any given system, as highlighted in this Review, arguably allows one to critically evaluate whether any predicted “Pd-free” methodology might operate at low ppm Pd levels. Caution is necessary, particularly when employing activated substrates and higher reaction temperatures (>60 °C).

## Supporting Information Available

Python ppm calculator documents (ZIP)
MATLAB app program documents (ZIP)
Cross-reference citation details and analysis (XLSX)

## Abbreviations

ppm: parts per million
DABCO: 1,4-diazabicyclo[2.2.2]octane
dmba: dimethyl benzyl amine
TON: turnover number
TOF: turnover frequency
DMF: dimethylformamide
DMA: dimethylacetamide
*JMC*: *Journal of Medicinal Chemistry*
NMP: *N*-methyl-2-pyrollidone
SMCC: Suzuki–Miyaura cross-coupling
LIMS: Laboratory Information Management Systems
