# Supplementary figures and images for: Pd-Catalyzed Cross-Couplings: On the Importance of the Catalyst Quantity Descriptors, mol % and ppm
Source: Org Process Res Dev. 2022 Jul 11;26(8):2240–69. doi: 10.1021/acs.oprd.2c00051 (PMC9396667; doi:10.1021/acs.oprd.2c00051)

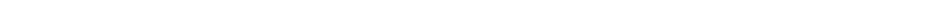

Supplement: Supplementary file 2 — op2c00051_si_002.zip [file op2c00051_si_002.zip › ppm_programs_updated/UoY_Line.png]
